# World-Wide Efficacy of Bone Marrow Derived Mesenchymal Stromal Cells in Preclinical Ischemic Stroke Models: Systematic Review and Meta-Analysis

**DOI:** 10.3389/fneur.2019.00405

**Published:** 2019-04-24

**Authors:** Nikunj Satani, Chunyan Cai, Kaavya Giridhar, Daryl McGhiey, Sarah George, Kaushik Parsha, Duyen M. Nghiem, Krystal S. Valenzuela, Jenny Riecke, Farhaan S. Vahidy, Sean I. Savitz

**Affiliations:** ^1^McGovern Medical School, Institute for Stroke and Cerebrovascular Diseases, The University of Texas Health Science Center at Houston, Houston, TX, United States; ^2^McGovern Medical School, Department of Internal Medicine, The University of Texas Health Science Center at Houston, Houston, TX, United States; ^3^Center for Clinical and Translational Sciences, McGovern Medical School, The University of Texas Health Science Center at Houston, Houston, TX, United States; ^4^Department of Neurology, McGovern Medical School, The University of Texas Health Science Center at Houston, Houston, TX, United States; ^5^Department of Psychology, The University of Texas Health Science Center at Austin, Austin, TX, United States; ^6^Department of Neurology, The University of Texas Southwestern Medical Center, Dallas, TX, United States

**Keywords:** ischemic stroke, meta-analysis, functional outcome, mesenchymal stromal cells, treatment effect, timing of administration, co-morbidities, gender differences

## Abstract

**Background:** Following extensive, positive results in pre-clinical experiments, Bone Marrow Derived-Mesenchymal Stromal Cells (BM-MSCs) are now being tested as a novel therapy for ischemic stroke in ongoing clinical trials. However, multiple critical questions relating to their translational application remain to be clarified. We performed a comprehensive, systematic review and meta-analysis of pre-clinical studies to evaluate the efficacy of BM-MSCs on functional outcomes after ischemic stroke, as well as the independent role of translational factors on their effect size.

**Methods:** We systematically reviewed the literature and identified articles using BM-MSCs in animal models of focal ischemic stroke. After abstraction of all relevant data, we performed a meta-analysis to estimate the combined effect size of behavioral endpoints after BM-MSC administration. To describe the effect size across many behavioral outcomes, we divided these outcomes into four categories: (1) Composite scores, (2) Motor Tests, (3) Sensorimotor Tests, and (4) Cognitive Tests. We also performed a meta-regression analysis for measuring the effect of individual characteristics of BM-MSC administration on the effect size.

**Results:** Our results from 141 articles indicate a significant beneficial effect on composite, motor, and sensorimotor outcomes after treatment with BM-MSCs compared to control groups. We found no major differences in treatment effect based on delivery route, dose, fresh vs. frozen preparation, or passage number. There were no consistent findings supporting a difference in treatment effect based on time windows from acute periods (0–6 h) vs. later windows (2–7 days). Furthermore, these positive treatment effects on functional outcome were consistent across different labs in different parts of the world as well as over the last 18 years. There was a negative correlation between publication year and impact factor.

**Conclusions:** Our results show worldwide efficacy of BM-MSCs in improving functional outcomes in pre-clinical animal models of stroke and support testing these cells in clinical trials in various ranges of time windows using different delivery routes. The continued growing number of publications showing functional benefit of BM-MSCs are now adding limited value to an oversaturated literature spanning 18 years. Researchers should focus on identifying definitive mechanisms on how BM-MSCs lead to benefit in stroke models.

## Introduction

Ischemic stroke is the 5th leading cause of death and the leading cause of long term disability in the United States (1, 2). Currently, tPA is the only FDA approved medical, non-invasive treatment for ischemic stroke, its use restricted by a narrow time window of 3 h (4.5 h in certain eligible patients) after symptom onset. This has limited the use of tPA to only a fraction of ischemic stroke patients.

Ischemic stroke results in a complex cascade of events leading to the loss of neural tissue, including neurons and their supporting structures (3, 4). Concurrently, there is an initiation of local and systemic inflammatory responses that evolve over a period of days, and the extent of which determines the eventual degree of damage (4). Considering the involvement of multiple parallel processes contributing to recovery, it is not surprising that stroke researchers have tested a multitude of investigational therapies for ischemic stroke with no significant advancement in stroke treatment.

Over the last two decades, Bone Marrow derived Mesenchymal Stromal Cells (BM-MSCs) have been investigated extensively as potential novel approach to promote recovery after ischemic stroke (5–7), and have advanced to clinical trials (8–10). BM-MSCs have demonstrated their beneficial effects by immunomodulation via multiple processes such as release of trophic factors, increasing angiogenesis, recruitment of neural precursors, synaptogenesis, as well as modulating immune responses from peripheral organs such as lungs and spleen (11, 12). Multiple preclinical animal studies have yielded encouraging results favoring the use of BM-MSCs for the treatment of ischemic stroke. These preclinical animal studies differ in various factors such as cell dose, timing, and route of administration, use of fresh or cultured cells, cell passage and the species from which the cells were isolated. In addition, the methodology of how and which outcomes are measured varies between different laboratories and researchers across the world.

As the use of BM-MSCs for ischemic stroke is translated to human clinical trials, there is a need to better understand the impact of individual, clinically relevant factors, as they most likely play a critical role in the potential treatment effects of BM-MSCs, and the design of clinical trials. Previously published meta-analyses have studied the results of preclinical studies using mesenchymal cells to evaluate their functional effect; however none of them extensively studied whether methodological variability between these preclinical trials affects functional outcome (13–15). Any reasonable interpretation of effect size, particularly with respect to timing of administration and delivery route was further limited by smaller sample size of studies included and behavioral tests they characterized. In addition, previous meta-analyses did not examine important clinically relevant variables such as gender of stroke animals, age of stroke animals, species from which MSCs were harvested, as well as the effect of stroke co-morbidities.

To address these issue, we conducted a comprehensive, updated, systematic review, and meta-analysis of all the preclinical publications between the years 2000 to 2018, which investigated the use of BM-MSCs to evaluate the efficacy of BM-MSCs in animal models of focal ischemic stroke. The primary aim of our analysis was to compare the effect size of behavioral improvement after BM-MSC administration vs. vehicle administration. To simplify the results from many behavioral tests in these articles, we divided these outcomes into four categories as previously described by our group (16): (1) Composite scores, (2) Motor Tests, (3) Sensorimotor Tests, and (4) Cognitive Tests. We also performed a meta-regression analysis to study whether heterogeneity among results of multiple studies is related to any specific characteristics of the treatment factors such as cell dose, labeling, timing and route of administration, time when outcome was measured, use of fresh or cultured cells, cell passage, species, and gender of animals from which cells were isolated, species and gender of stroke animals, co-morbidities as well as the laboratory where study was conducted.

## Materials and Methods

### Search Strategy

**Figure 1:**
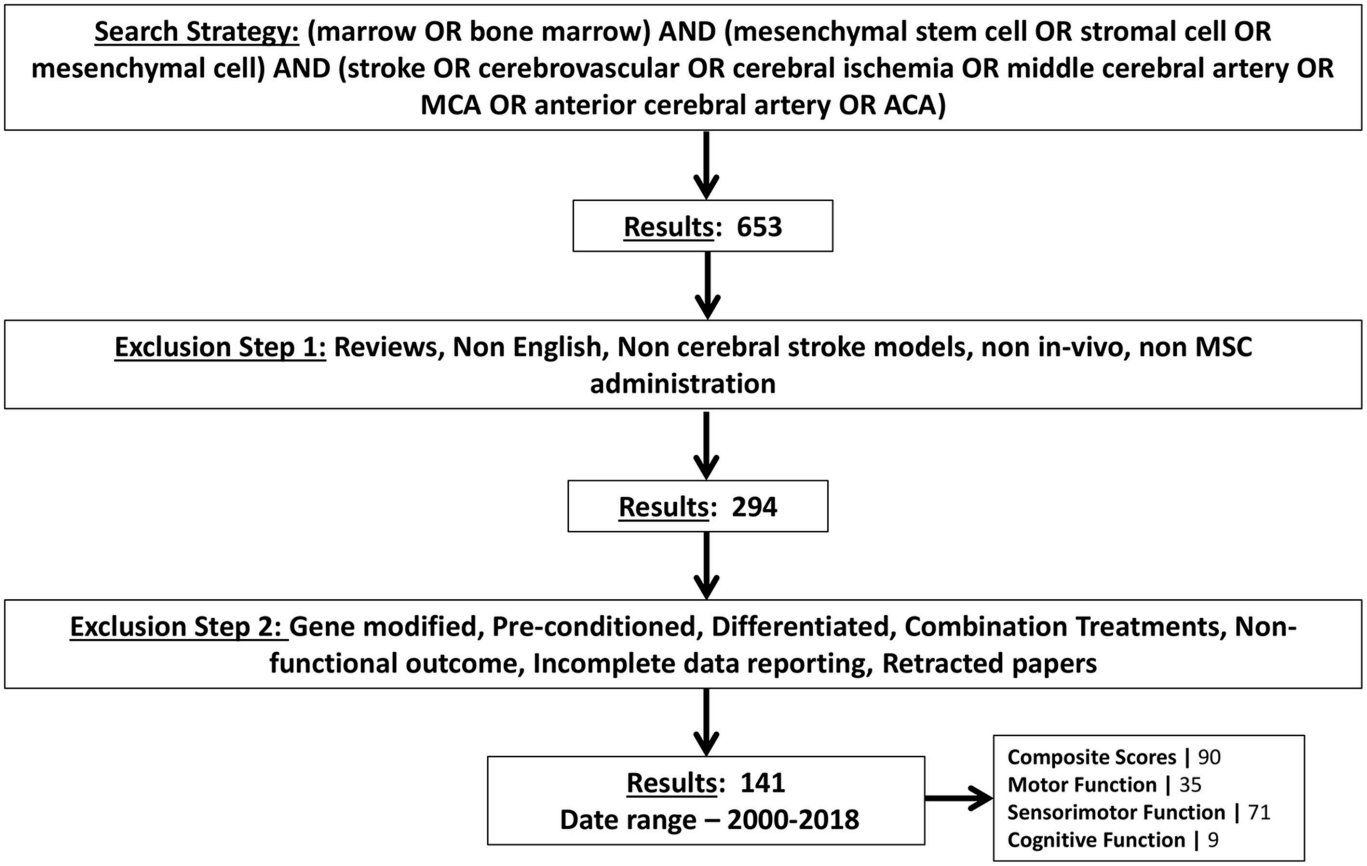
Schematic showing the search strategy for selecting articles for meta-analysis. Exclusion criteria are also shown. Number of articles found by this search strategy are shown and further stratified by type of functional tests they measured.

Previous reports investigating the use of BM-MSCs in ischemic stroke were identified by an electronic search of PUBMED and Google Scholar using the following search terms: “marrow OR bone marrow” AND “mesenchymal stem cell OR stromal cell OR mesenchymal cell” AND “stroke OR cerebrovascular OR cerebral ischemia OR middle cerebral artery OR MCA OR anterior cerebral artery OR ACA.” We excluded all reports which did not study functional outcome. In order to be maximally relevant to human clinical trials, studies investigating the use of gene modified BM-MSCs were also excluded. Studies in languages other than English were excluded. All other exclusion criteria are shown in Figure 1. A total of 141 articles were selected using the search strategy (Figure 1) and data were subsequently extracted. The final search was carried out on December 12, 2018.

### Inclusion Criteria

Published studies were included if they fulfilled all of the following criteria: (1) assessed bone marrow derived mesenchymal stromal cells in animal models of focal ischemic stroke; and (2) reported functional outcomes.

### Exclusion Criteria

We excluded review articles, editorials, commentaries, letters which reported no new data, meta-analyses as well as abstracts. In addition, we excluded the following articles that: (1) were not written in English; (2) studied non-focal stroke models; (3) had no *in-vivo* experiments; (4) did not use bone marrow derived mesenchymal stromal cells; (5) used gene-modified BM-MSCs; (6) used preconditioned BM-MSCs; (7) used differentiated BM-MSCs; (8) used BM-MSCs with other drugs or cells as combination treatments; (9) did not assess functional outcomes; (10) did not report SD or SE; (11) had SD or SE as 0 (zero); and (12) did not report sample size.

### Data Extraction

After running the search strategy and applying inclusion and exclusion criteria, we found 141 articles. We extracted data of all functional outcomes measured in these studies. We collected functional endpoints from all tables and the results. For line graphs, the data were extracted from the graphics using Web Plot Digitizer Version 4.1 - (https://automeris.io/WebPlotDigitizer/index.html). Two independent abstractors collected each outcome. Average of the data collected from the two users was used to run statistical analysis.

We collected data on behavioral tests conducted in these articles and divided them into four categories: (1) Composite Scores—which included Neurological severity score, Garcia Score, Roger Scale, Bederson Score, and Longa Score; (2) Motor Tests—which included cylinder test, foot fault test, limb placement, beam balance test, tightrope test and grid walking test; (3) Sensorimotor tests—which included adhesive removal test, treadmill stress test, rotarod test, corner test, elevated body swing test and limb stride length measurement; and (4) Cognitive tests—which included water maze test, eight arm radial maze test and novel object recognition test. We used this classification of functional outcomes as per our previously published paper (16). Considering the comprehensive nature of our meta-analysis, this classification allowed us to analyze and organize meta-analysis and meta-regression data from all functional tests and provided a guide for future pre-clinical and clinical trials assessing functional data.

In addition to collecting the functional outcomes, we also collected information about the BM-MSCs used to administer in these articles. We collected information on variables such as cell passage, cell labeling, time when functional outcome was measured, time when cells were administered, dosage of cells used, whether the cells were used fresh or cryopreserved before use, donor and species of BM-MSCs, the laboratory where the experiments were conducted. These variables were categorized as per Table 1. Only the last time point measurements were examined for behavioral testing if outcomes were reported at multiple time points within the same experiment. When there were multiple independent experiments in one article, we included all of these experiments into the analysis. For calculating dose of BM-MSCs, we took the average reported weight of stroke animals and converted all doses to cells per kilogram. We also analyzed effect sizes based on year of article publication. We calculated 25, 50, and 75 th quartiles of the publication year and categorized experiments into four groups based on publication year: 2000–2008, 2009–2012, 2013–2015, and 2016–2018.

**Table 1:** List of all variables collected in this meta-analysis.

**Variables collected from 141 articles**
**Cell labeling**
Yes | No
**Route of administration**
Intra-arterial | Intravenous | Intracranial
**Time when outcome was measured**
<2 weeks | 2–4 weeks | 4–12 weeks | >12 weeks
**Dose of bm-mscs administered**
>1*10E6 | <= 1*10E6
**Time of bm-msc Administration**
0–6 h vs. > 7 Days
12–24 h vs. >7 Days
2–7 Days vs. >7 Days
0–6 h vs. 2–7 Days
12–24 h vs. 2–Days
0–6 h vs. 12–24 h
**Were bm-mscs cryopreserved or not?**
Fresh | Frozen
**Passage of administered bm-mscs**
2–4 vs. >4
**Species of bm-msc donor**
Rat | Mouse | Human
**Gender oF bm-msc donor**
Female|Male | Unknown
**Species of stroke animal**
Rat | Mouse | Rabbit | Monkey | Dog
**Gender of stroke animal**
Female | Male | Unknown
**Age of stroke animal**
Adult | Retired Breeder
**Stroke animal co-morbidities**
Normal | T1DM | T2DM | SCID
**Continent**
Asia | North America | Europe | South America
**Year published**
2000–2008 | 2009–2012 | 2013–2015 | 2016–2018
**Impact factor of journal where article was published**

### Statistical Analysis

The effect size of BM-MSCs therapy was calculated as the standardized mean difference (SMD) of these interested outcomes between MSC and Vehicle-treated groups based on Hedges' method. The overall heterogeneity was examined by I^2^ and Cochran's Q-statistic test (17). A *p* < 0.1 was considered statistically significant for the Cochran's Q-statistic test (18). Since heterogeneity exists for all four types of outcomes, random effects model using DerSimonian and Laird method was applied to obtain the pooled effect size. We multiplied the outcomes by-−1 for larger values, indicating superior outcome if needed. For each type of outcome, we generated forest plots to depict the SMD along with its 95% confidence interval (CI) for each individual experiment as well as the pooled SMD of all studies. The statistical significance of the pooled effect size was performed by z-test. To confirm whether our findings were driven by any single study, a leave-one-out sensitivity analysis was performed by iteratively removing one study at a time. Excessive influence was suspected if the main estimate of omitting an individual study lied outside the 95% CI of the combined analysis.

Meta regression analysis was performed to assess whether heterogeneity among results of multiple studies is related to any specific characteristics of the studies, if the significance of heterogeneity was found. Univariable meta-regression analyses based on a random effects model with restricted maximum likelihood estimation were performed for four types of outcomes. Potential publication bias was evaluated using funnel plots and Egger test was performed to evaluate the symmetry of the funnel plots (19). If asymmetry was observed, a Trim and Fill procedure (20) was applied to identify the existence of unpublished hidden studies. By imputing the presence of these potential missing studies, an adjusted pooled estimate was provided. All analyses were performed with StataMP 11 (StataCorp LP, College Station, TX).

## Results

### Study Characteristics

The flow diagram shows that the initial search yielded 653 pubmed articles. After applying all exclusion criteria, we found 141 articles and relevant data was subsequently extracted. The number of articles identified and excluded at each step are depicted in Figure 1. Among all articles measuring functional outcomes, 90 articles reported composite scores, 35 articles reported motor outcomes, 71 articles reported sensorimotor outcomes, and 9 articles reported cognitive outcomes. Some articles conducted multiple independent, behavioral tests to measure outcome in the same category. Hence, a total of 105 experiments reported composite scores, 53 experiments reported motor function, 101 experiments reported sensorimotor function and 10 experiments reported cognitive function. Frequency and percentage of all interested variables by test categories was calculated in SAS (Table S1).

### Treatment Effect Size

#### Composite Score

**Figure 2:**
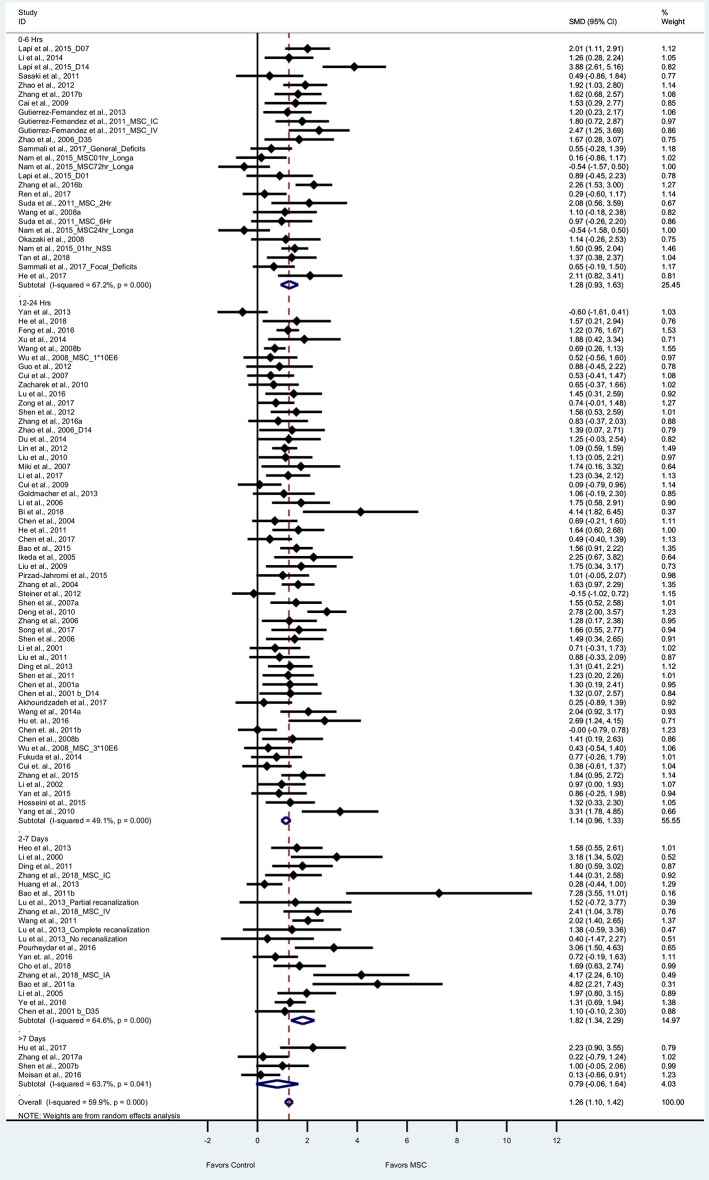
Forest plot showing standardized mean difference of composite score between BM-MSC therapy and control groups, stratified by timing of administration of BM-MSCs. 95% confidence intervals are shown for all studies measuring composite scores. 95% confidence intervals are further stratified by timing of administration of BM-MSCs into 0–6 h, 12–24 h, 2–7 days, and >7 days. *p*-values are for heterogeneity measured by Cochran's Q-statistic test, values <0.1 are significant.

We performed a meta-analysis to evaluate the effect of BM-MSCs on overall neurological function, by assessing composite scores from 90 articles (105 experiments) based on tests of mNSS, Bederson score, Longa Score, and Roger scale. These tests are routinely used by different researchers around the world to assess severity of neurological damage after stroke. For the composite score, the pooled effect size of BM-MSC therapy was substantial and significant (1.26, 95% CI: 1.10–1.42), which demonstrates a significant decrease in the composite score in the BM-MSC group compared with vehicle treatment (Figure 2). Random effects model was applied since significant heterogeneity was observed by Cochran's Q-statistic test (*p* < 0.0001). Furthermore, we stratified these experiments by timing of BM-MSC therapy: 0–6 h, 12–24 h, 1–7days and >7days (Figure 2). We found that the pooled effect size in each of these groups remained significant (*p* < 0.0001) except for >7days (*p* = 0.07).

#### Motor Function

**Figure 3:**
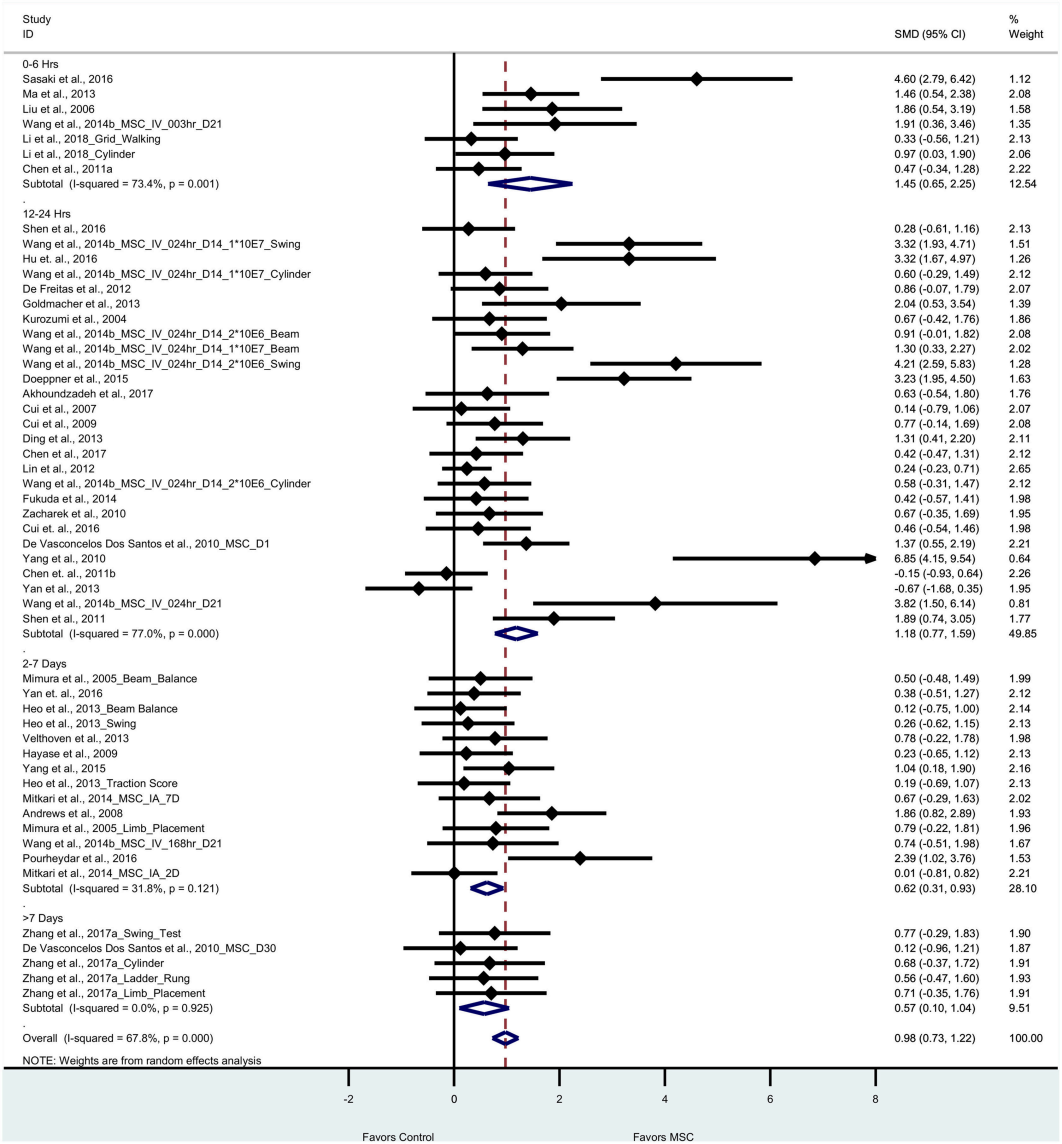
Forest plot showing standardized mean difference of motor function between BM-MSC therapy and control groups, stratified by timing of administration of BM-MSCs. 95% confidence intervals are shown for all studies measuring motor function. 95% confidence intervals are further stratified by timing of administration of BM-MSCs into 0–6 h, 12–24 h, 2–7days, and >7 days. *p*-values are for heterogeneity measured by Cochran's Q-statistic test, values <0.1 are significant.

Thirty five articles (53 experiments) reported motor outcomes based on tests that evaluate motor deficits such as cylinder test, beam walking test, elevated body swing test, foot fault, ladder-rung walking test, and grid walking test. For motor outcomes, the pooled effect size of BM-MSC therapy was substantial and significant as compared to vehicle group (0.98, 95% CI: 0.73–1.22), which demonstrates that BM-MSCs significantly improved motor outcomes (Figure 3). Random effects model was applied since significant heterogeneity was observed by Cochran's Q-statistic test (*p* < 0.0001). When we stratified motor outcomes by timing of treatment, we found that BM-MSCs significantly improved outcomes for treatment at 0–6 h, 12–24 h, and 2–7days (*p* < 0.0001 for all three groups, Figure 3). BM-MSC treatment at >7 days also improved motor outcomes (*p* = 0.02).

#### Sensorimotor Function

**Figure 4:**
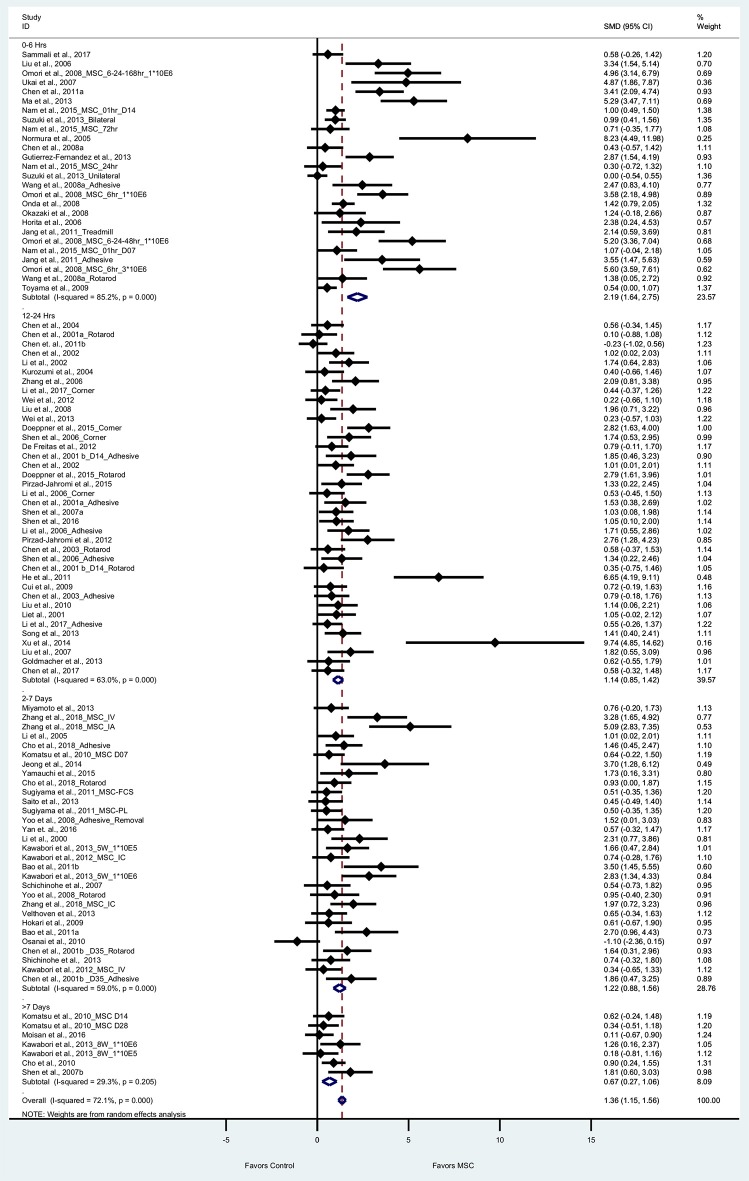
Forest plot showing standardized mean difference of sensorimotor function between BM-MSC therapy and control groups, stratified by timing of administration of BM-MSCs. 95% confidence intervals are shown for all studies measuring sensorimotor function. 95% confidence intervals are further stratified by timing of administration of BM-MSCs into 0–6 h, 12–24 h, 2–7 days, and >7 days. *p*-values are for heterogeneity measured by Cochran's Q-statistic test, values <0.1 are significant.

Seventy one articles (101 experiments) reported sensorimotor outcomes as measured by tests such as adhesive removal, rotarod, corner, and treadmill test. The pooled effect size of BM-MSC therapy based on sensorimotor outcomes was substantial and significant (1.36, 95% CI: 1.15–1.56), which demonstrates the significant increase of sensorimotor outcome in the BM-MSC group compared with vehicle treatment (Figure 4). Random effects models were applied due to significant heterogeneity assessed by Cochran's Q-statistic test. When we stratified sensorimotor outcomes based on timing of BM-MSC therapy, we found that BM-MSCs significantly improved outcomes for all treatment time-groups (*p* < 0.0001 for 0–6 h, 12–24 h, and 2–7days; *p* = 0.001 for >7day BM-MSC treatment, Figure 4).

#### Cognitive Function

**Figure 5:**
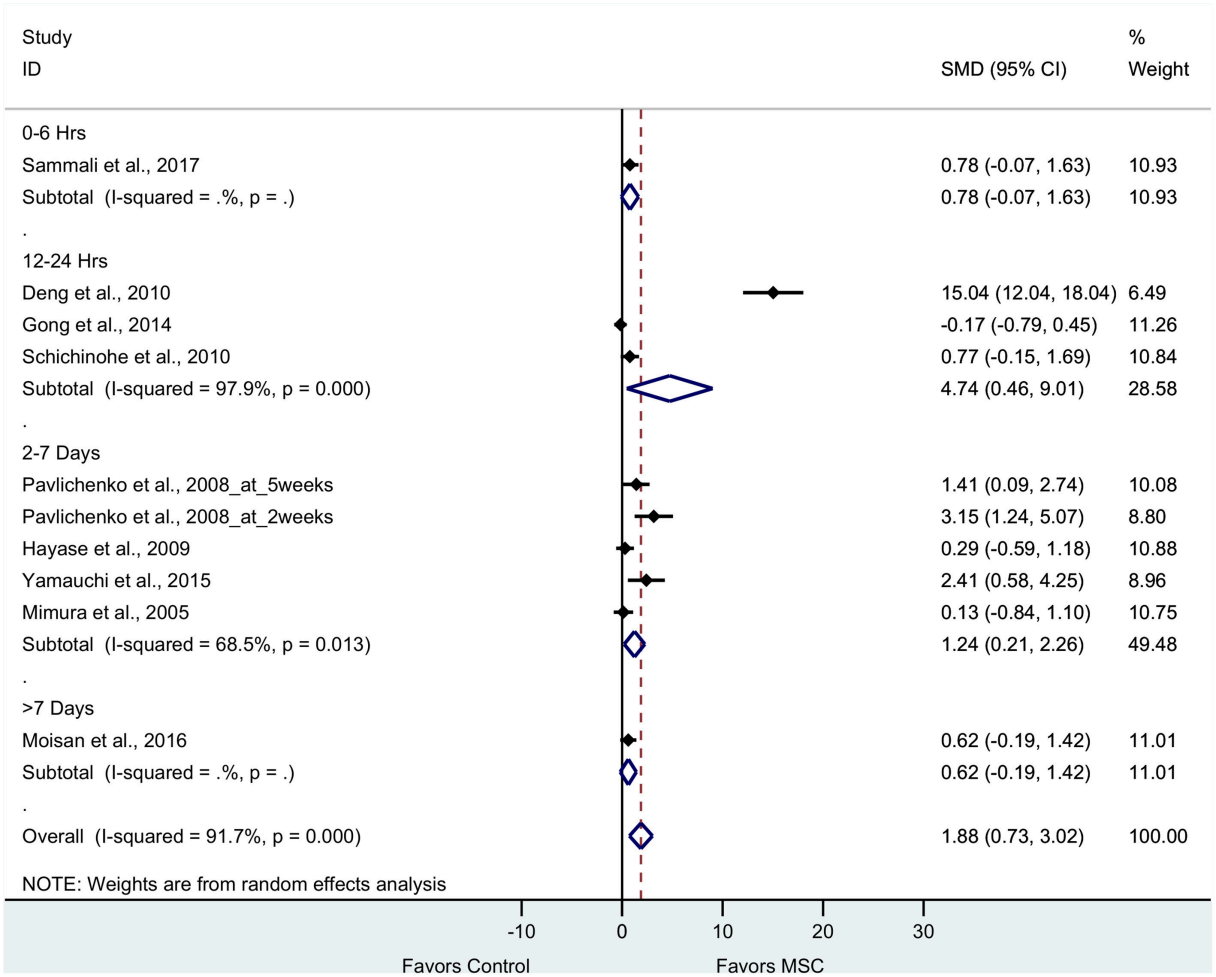
Forest plot showing standardized mean difference of cognitive function between BM-MSC therapy and control groups, stratified by timing of administration of BM-MSCs. 95% confidence intervals are shown for all studies measuring cognitive function. 95% confidence intervals are further stratified by timing of administration of BM-MSCs into 0–6 h, 12–24 h, 2–7 days, and >7 days. *p*-values are for heterogeneity measured by Cochran's Q-statistic test, values <0.1 are significant.

Nine articles (10 experiments) reported cognitive outcomes from tests such as water maze, radial maze and novel object recognition tests. The pooled effect size of BM-MSC therapy based on cognitive function was substantial and significant (1.88, 95% CI: 0.73–3.02, *p* = 0.001), which demonstrated significant increase of cognitive function in the BM-MSC group compared with vehicle treatment (Figure 5). Random effects models were applied due to significant heterogeneity assessed by Cochran's Q-statistic test (*p* < 0.0001).

### Meta-Regression

Cochran's Q-statistic test for heterogeneity suggested a significant effect of BM-MSCs for all four outcomes (*p* < 0.0001). We then performed univariable meta-regression analysis for composite score, motor function, and sensorimotor function to assess whether heterogeneity among results of multiple studies is related to any specific characteristics in these studies. Frequency and percentage of all interested variables by test categories are reported in Table S1. We did not perform meta-regression analysis for cognitive function due to the limited number of studies. The regression coefficients in Tables 2–**4** estimate how the effect of BM-MSCs on these outcomes in each subgroup differs from the reference group.

**Table 2:** Univariable meta-regression analysis to assess the impact of study variables related to MSC therapy on composite score.

**Variables collected**	**Comparison**	**Coefficients**	**Standard error**	***p*-value**
Cell labeling	Yes vs. No	0.057	0.182	0.76
Route of administration	IA vs. IC	0.309	0.326	0.35
	IV vs. IC	−0.162	0.212	0.45
	IV vs. IA	−0.471	0.288	0.11
Time when outcome was measured	<2 weeks vs. >12 weeks	−0.262	0.540	0.63
	2–4 weeks vs. >12 weeks	0.049	0.554	0.93
	4–12 weeks vs. >12 weeks	0.097	0.576	0.87
	<2 weeks vs. 4–12 weeks	−0.359	0.256	0.16
	2–4 weeks vs. 4–12 weeks	−0.049	0.285	0.97
	<2 weeks vs. 2–4 weeks	−0.311	0.204	0.13
Cell dose (Total cells)	>1*10E6 vs. <= 1*10E6	−0.109	0.180	0.55
Cell dose (per kilogram)	Cells/kg weight of stroke animal	−8.69*10^−9^	8.62*10^−9^	0.32
Timing of BM-MSC administration	0–6 h vs. > 7 Days	0.499	0.454	0.28
	12–24 h vs. >7 Days	0.378	0.437	0.39
	2–7 Days vs. >7 Days	0.973	0.476	**0.04**
	0–6 h vs. 2–7 Days	−0.474	0.276	**0.09**
	12–24 h vs. 2–7 Days	−0.595	0.247	**0.02**
	0–6 h vs. 12–24 h	0.121	0.203	0.55
Fresh/Frozen BM-MSCs	Fresh vs. Frozen	0.349	0.206	**0.09**
Passage of BM-MSCs	2–4 vs. >4	0.051	0.257	0.84
	Unknown vs. >4	0.015	0.298	0.96
	Unknown vs. 2–4	−0.036	0.217	0.87
Species of BM-MSC donor	Mouse vs. Human	0.276	0.451	0.54
	Rat vs. Human	0.395	0.205	**0.06**
	Dog vs. Human	0.086	0.762	0.91
	Mouse vs. Rat	−0.119	0.428	0.78
	Dog vs. Rat	−0.309	0.748	0.68
Donor gender	Female vs. Male	−0.364	0.357	0.31
	Unknown vs. Male	−0.069	0.193	0.72
	Unknown vs. Female	0.295	0.339	0.39
Gender of stroke animal	Female vs. Male	0.275	0.329	0.41
	Unknown vs. Male	0.140	0.310	0.65
	Unknown vs. Female	−0.134	0.431	0.76
Species of stroke animal	Mice vs. Rat	−0.525	0.320	0.10
Age of stroke animal	Adult vs. Retired Breeder	−0.211	0.402	0.60
Co–morbidities	Normal vs. Non-normal	0.820	0.384	**0.04**
Continent	Asia vs. North America	0.394	0.190	**0.04**
	Europe vs. North America	0.412	0.321	0.20
	Europe vs. Asia	0.018	0.302	0.95
Year	2009–2012 vs. 2000–2008	0.202	0.256	0.43
	2013–2015 vs. 2000–2008	−0.123	0.252	0.630
	2016–2018 vs. 2000–2008	0.056	0.244	0.82
	2013–2015 vs. 2009–2012	−0.325	0.252	0.2
	2016–2018 vs. 2009–2012	−0.146	0.244	0.55
	2016–2018 vs. 2013–2015	0.179	0.239	0.46

*Positive coefficients indicate a larger MSC effect. Bold values indicate significance (p < 0.05) or showing a trend toward significance (p < 0.1)*.

#### Route of Administration

There was no difference in treatment effect on improving composite scores and sensorimotor function regardless of route of BM-MSC administration (Tables 2, 4). However, intravenously administered BM-MSCs showed significantly more treatment effect in improving motor outcomes as compared with intra-cranial injections (*p* = 0.04, Table 3).

**Table 3:** Univariable meta-regression analysis to assess the impact of study variables related to MSC therapy on motor function.

**Variables collected**	**Comparison**	**Coefficients**	**Standard error**	***p*-value**
Cell labeling	Yes vs. No	−0.208	0.350	0.55
Route of administration	IA vs. IC	−0.203	0.659	0.76
	IV vs. IC	0.710	0.331	**0.04**
	IV vs. IA	0.913	0.630	0.15
Time when outcome was measured	<2 weeks vs. 4–12 weeks	0.187	0.468	0.69
	2–4 weeks vs. 4–12 weeks	0.171	0.497	0.73
	<2 weeks vs. 2–4 weeks	0.016	0.362	0.97
Cell dose (Total cells)	>1*10E6 vs. <= 1*10E6	−0.128	0.313	0.69
Cell dose (per kilogram)	Cells/kg weight of stroke animal	1.94*10^−9^	8.84*10^−9^	0.83
Timing of BM-MSC administration	0–6 h vs. > 7 Days	0.852	0.662	0.20
	12–24 h vs. >7 Days	0.583	0.545	0.29
	2–7 Days vs. >7 Days	0.103	0.578	0.86
	0–6 h vs. 2–7 Days	0.749	0.523	0.16
	12–24 h vs. 2–7 Days	0.480	0.364	0.19
	0–6 h vs. 12–24 h	0.269	0.486	0.58
Fresh/Frozen BM-MSCs	Fresh vs. Frozen	0.147	0.365	0.69
Passage of BM-MSCs	2–4 vs. >4	−1.030	0.357	**0.006**
	Unknown vs. >4	−0.452	0.432	0.30
	Unknown vs. 2–4	0.575	0.357	0.11
Species of BM-MSC donor	Mouse vs. Human	0.165	0.657	0.80
	Rat vs. Human	0.199	0.377	0.60
	Mouse vs. Rat	−0.034	0.602	0.96
Donor gender	Unknown vs. Male	0.067	0.322	0.84
Gender of stroke animal	Unknown vs. Male	−0.175	0.601	0.77
Species of stroke animal	Mice vs. Rat	0.452	0.484	0.36
Co-morbidities	Normal vs. Non-normal	0.583	0.521	0.27
Continent	Asia vs. North America	−0.278	0.345	0.42
	Europe vs. North America	−0.079	0.618	0.90
	South America vs. North America	−0.330	0.689	0.63
	Asia vs. Europe	−0.199	0.623	0.75
	South America vs. Europe	−0.251	0.863	0.77
	Asia vs. South America	0.052	0.693	0.94
Year	2009–2012 vs. 2000–2008	−0.100	0.585	0.87
	2013–2015 vs. 2000–2008	0.168	0.535	0.76
	2016–2018 vs. 2000–2008	0.064	0.568	0.91
	2013–2015 vs. 2009–2012	0.268	0.426	0.53
	2016–2018 vs. 2009–2012	0.163	0.466	0.73
	2016–2018 vs. 2013–2015	−0.104	0.401	0.80

*Positive coefficients indicate a larger MSC effect. Bold values indicate significance (p < 0.05) or showing a trend toward significance (p < 0.1)*.

**Table 4:** Univariable meta-regression analysis to assess the impact of study variables related to MSC therapy on sensorimotor function.

**Variables collected**	**Comparison**	**Coefficients**	**Standard error**	***p*-value**
Cell labeling	Yes vs. No	−0.426	0.284	0.14
Route of administration	IA vs. IC	0.940	0.558	0.10
	IV vs. IC	0.473	0.302	0.12
	IV vs. IA	−0.467	0.530	0.38
Time when outcome was measured	<2 weeks vs. >12 weeks	0.312	0.758	0.68
	2–4 weeks vs. >12 weeks	1.008	0.776	0.20
	4–12 weeks vs. >12 weeks	0.261	0.760	0.73
	<2 weeks vs. 4–12 weeks	0.051	0.312	0.87
	2–4 weeks vs. 4–12 weeks	0.748	0.353	**0.04**
	<2 weeks vs. 2– weeks	−0.696	0.349	**0.049**
Cell dose (Total cells)	>1*10E6 vs. <= 1*10E6	0.268	0.273	0.33
Cell dose (per kilogram)	Cells/kg weight of stroke animal	3.35*10^−8^	1.98*10^−8^	**0.09**
Timing of BM-MSC administration	0–6 h vs. > 7 Days	1.361	0.536	**0.01**
	12–24 h vs. >7 Days	0.476	0.508	0.35
	2–7 Days vs. >7 Days	0.567	0.523	0.28
	0–6 h vs. 2–7 Days	0.794	0.362	**0.03**
	12–24 h vs. 2–7 Days	−0.091	0.320	0.78
	0–6 h vs. 12–24 h	0.885	0.339	**0.01**
Fresh/Frozen BM-MSCs	Fresh vs. Frozen	−0.700	0.302	**0.02**
	Unknown vs. Frozen	−1.304	0.533	**0.02**
	Unknown vs. Fresh	−0.604	0.495	0.23
Passage of BM-MSCs	2–4 vs. >4	−0.832	0.365	**0.03**
	Unknown vs. >4	−0.328	0.403	0.42
	Unknown vs. 2–4	0.504	0.306	0.10
Species of BM-MSC donor	Mouse vs. Human	−0.417	0.683	0.54
	Rat vs. Human	−0.486	0.296	0.10
	Mouse vs. Rat	0.069	0.660	0.92
Donor gender	Female vs. Male	−0.211	0.820	0.8
	Unknown vs. Male	−0.003	0.325	0.99
	Unknown vs. Female	0.208	0.787	0.79
Gender of stroke animal	Female vs. Male	0.897	0.406	**0.03**
	Unknown vs. Male	−0.147	0.481	0.76
	Unknown vs. Female	−1.044	0.593	**0.08**
Species of stroke animal	Mice vs. Rat	0.039	0.441	0.93
Age of stroke animal	Adult vs. Retired Breeder	−0.076	0.633	0.91
Continent	Asia vs. North America	0.453	0.291	0.12
	Europe vs. North America	0.413	0.586	0.48
	Asia vs. Europe	0.040	0.571	0.94
Year	2009–2012 vs. 2000–2008	−0.544	0.355	0.13
	2013–2015 vs. 2000–2008	−0.276	0.348	0.43
	2016–2018 vs. 2000–2008	−0.500	0.439	0.26
	2013–2015 vs. 2009–2012	0.268	0.390	0.49
	2016–2018 vs. 2009–2012	0.043	0.473	0.93
	2016–2018 vs. 2013–2015	−0.225	0.467	0.63

*Positive coefficients indicate a larger MSC effect. Bold values indicate significance (p < 0.05) or showing a trend toward significance (p < 0.1)*.

#### Dose of BM-MSCs

There was no difference in treatment effect on all functional outcomes regardless of the dose of BM-MSCs used (Tables 2–4 and Figure S1). There was a trend of an inverse correlation between dose (cells/kg) and treatment effect on composite scores. There was also a trend of direct correlation between dose (cells/kg) and treatment effect on sensorimotor function. However, none of these trends reached statistical significance. BM-MSCs produced significant improvement in all functional outcomes regardless of dose.

#### Timing of Administration

When BM-MSC were administered between 2 and 7 days, they significantly improved composite scores as compared to 12–24 h and >7days (*p* = 0.02 and 0.04, respectively). There was marginal significance on improvement of composite score for 2–7 days administration as compared with 0–6 h (*p* = 0.09, Table 2). BM-MSCs showed similar treatment effect in improving motor function regardless of timing of administration (Table 3). In addition, a significant difference in treatment effect size on sensorimotor function was found when BM-MSC were administered between 0 and 6 h as compared with 12–24 h (*p* = 0.01), 2–7days (*p* = 0.03), or >7 days (*p* = 0.01) (Table 4). The pooled effect size when BM-MSCs were administered after 7 days of ischemic stroke showed smaller significance as compared to when they were administered before 7 days (*p* = 0.07, 0.02, and 0.001 for composite score, motor function and sensorimotor function, respectively). Furthermore, when BM-MSCs were administered after 4 weeks, the forest plots do show benefit. However, the effect size was significantly less and inconsistent across studies (Figure S2).

#### Gender and Age Differences

BM-MSCs improved functional endpoints on all outcomes for both male and female stroke animals. However, female animals showed significantly more effect in improving sensorimotor function after BM-MSC treatment as compared to male animals (*p* = 0.03, Table 4). There was no such difference in treatment effect on improving composite scores or motor function. The pooled effect size for both composite score and sensorimotor function was significant in young as well as old stroke animals (*p* < 0.0001 for all groups, Table S2). However, the 95% confidence interval in old animals was wider due to limited sample size (*N* = 5).

#### Co-morbidities

Although BM-MSCs improved outcomes in animals with and without comorbidities. Stroke animals without comorbidities showed significantly more improvement in composite scores after MSC treatment as compared to stroke animals with co-morbidities such as Type 1 Diabetes Mellitus (T1DM) or Type 2 Diabetes Mellitus (T2DM) (*p* = 0.04, Table 2).

#### BM-MSC Characteristics (Labeling, Donor Species, and Gender)

Labeling BM-MSCs did not change the efficacy of BM-MSCs on improving composite score, motor function and sensorimotor function. Furthermore, regardless of the species and gender of the donor of BM-MSCs, there was similar improvement in functional outcomes across all three categories. (Tables 2–4)

Fresh vs. Frozen BM-MSCs: Both fresh and frozen BM-MSCs had similar treatment effect in improving composite score and motor function (Tables 2, 3). However, frozen BM-MSCs produced significantly improved sensorimotor outcome as compared with fresh BM-MSCs (*p* = 0.02, Table 4).

#### Passage of BM-MSCs

BM-MSCs improved functional outcomes regardless of the passage of cells used. There was no significant difference in treatment effect on improvement of composite scores whether cell passage was <4 or >4 (Table 2). BM-MSCs with >4 passages produced significant improvement in motor outcomes compared with passages <4 (*p* = 0.006, Table 3). BM-MSC with >4 passages also produced significant improvement in sensorimotor outcomes as compared to BM-MSC at 2–4 passages (*p* = 0.03, Table 4).

#### Laboratories

Laboratories from around the world showed significant improvement in all functional outcomes after BM-MSC treatment for ischemic stroke. On the composite outcome score, the effect size was more pronounced in studies from Asia as compared with North America (*p* = 0.04, Table 2).

#### Year of Publication

We calculated 25, 50, and 75 th quartiles of the publication year and categorized experiments into four groups based on publication year: 2000–2008, 2009–2012, 2013–2015, and 2016–2018. Publications from all four groups showed improvement in functional outcomes after BM-MSC administration with no significant difference between the groups.

#### Journal Impact Factor

**Figure 6:**
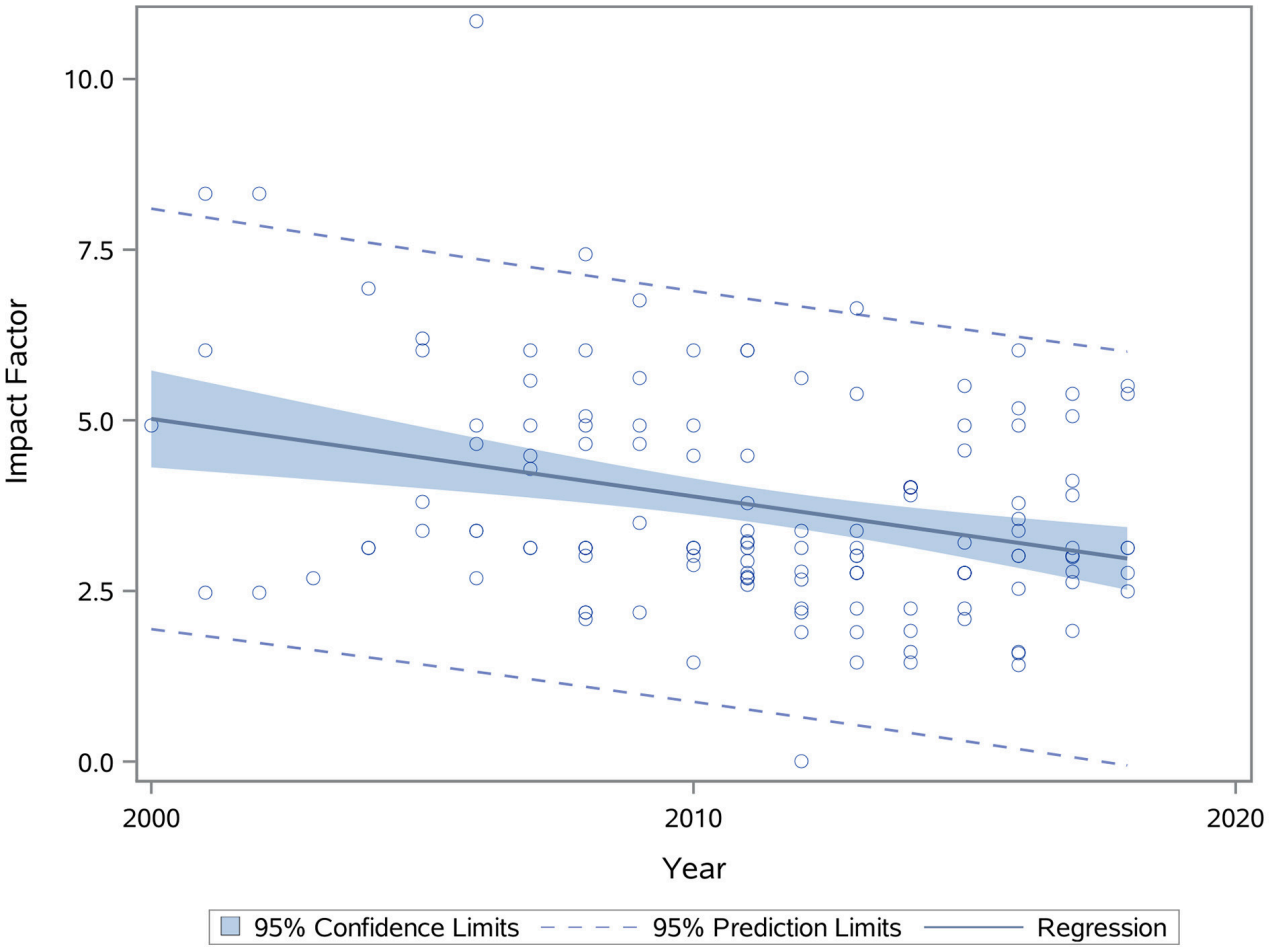
Scatterplot showing relationship between journal impact factor and year of publication (*p* = 0.005). Spearman correlation coefficient was calculated to find correlation between these two variables. *p* < 0.05 is significant.

We performed an analysis of impact factor since 2000 for all articles reporting functional outcomes after BM-MSC treatment. We observed a negative correlation between year and impact factor (Spearman correlation coefficient: −0.23, *p* = 0.005, Figure 6).

#### Sensitivity Analysis

To evaluate the robustness of the calculated pooled effect size, we performed a leave-one-out sensitivity analysis by iteratively removing one study at a time and recalculating the pooled effect size of the remaining studies for each test category. For composite scores, motor function, and sensorimotor function, the pooled effect size after removing each one of the studies was stable indicating that our results were not driven by any single study. However, for cognitive function, the pooled effect size after removing the experiment in Deng et al. (21) reduced from 1.88 (95% CI: 0.73–3.02) to 0.74 (95% CI: 0.22–1.26) and statistically significant (*p* = 0.005).

#### Publication Bias

**Figure 7:**
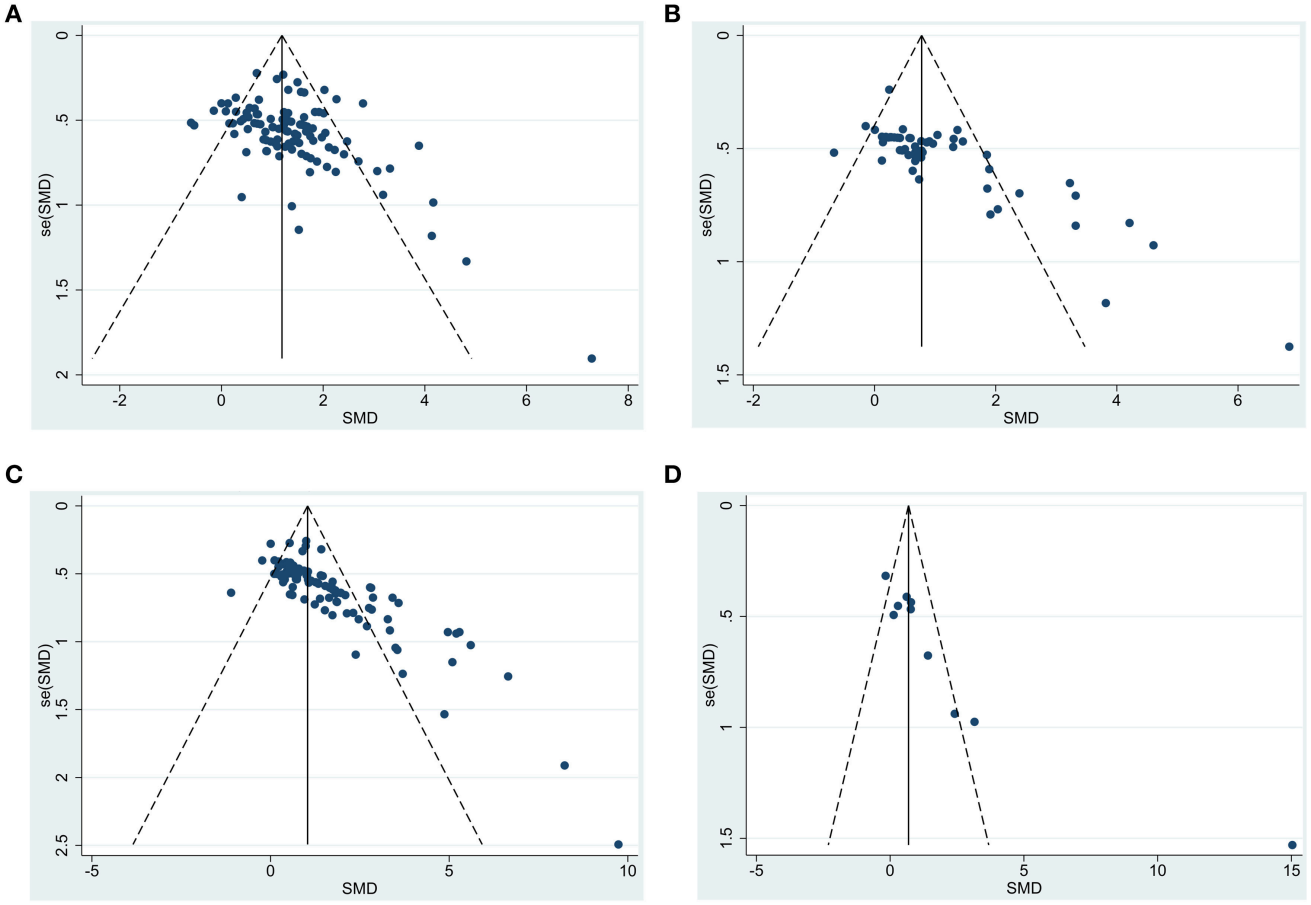
Funnel plot to detect publication bias for functional outcomes after BM-MSC administration. **(A)** funnel plot to detect publication bias in all studies measuring composite scores; **(B)** funnel plot to detect publication bias in all studies measuring motor function; **(C)** funnel plot to detect publication bias in all studies measuring sensorimotor function; **(D)** funnel plot to detect publication bias in all studies measuring cognitive function.

Based on the funnel plots (Figure 7), we observed significant publication bias for the outcomes for composite scores, cognitive function, motor function, and sensorimotor function (*p* = 0.002, 0.002, < 0.0001, and <0.0001, respectively). By trim and fill approach, for composite score, we added 25 unpublished hidden experiments and obtained the adjusted pooled effect size at 0.96 (95% CI: 0.78–1.13) after accounting for publication bias, which is still statistically significant (*p* < 0.0001). Similarly, for motor function and sensorimotor function, we added 10 and 30 unpublished hidden experiments and obtained the adjusted pooled effect size at 0.63 (95% CI: 0.34–0.92) and 0.80 (95% CI: 0.56–1.03) after accounting for publication bias. For cognitive function, after trim and fill approach, we added 4 unpublished hidden experiments and obtained the adjusted pooled effect size at 0.37 (95% CI: −0.92 to 1.65), which led to statistically non-significant results on cognitive function (p = 0.58).

## Discussion

### Robust Efficacy Across Outcomes and Laboratories

We have performed a comprehensive assessment of preclinical studies testing the efficacy of BM-MSCs in preclinical stroke models. BM-MSCs were exclusively chosen because they are the most widely studied cell therapy in animal stroke models and BM-MSCs differ in their characteristics compared with MSCs derived from other tissue sources (22–24). Based on our meta-analysis of 141 articles, we found that BM-MSCs had broad treatment effects on a number of different functional outcomes that included composite scores, motor function, sensorimotor function, and cognitive function. Many prior meta-analyses have examined functional outcomes after BM-MSC therapy (13–15). We conducted a comprehensive meta-analysis to analyze all published articles in English from 2000 to 2018 and provided updated data on various clinically relevant factors. To provide more in-depth analyses, we included all behavioral tests from qualifying studies and grouped them into four categories based on our prior published work (16). We found significant improvement with a high mean effect size in all four categories of functional outcome, suggesting a robust effect across all outcomes. These effect sizes remained significant after adjusting for publication bias for all outcomes except cognitive outcome. Positive effects on cognitive outcomes appeared driven by one study. It is possible that BM-MSCs do not improve cognitive outcome or the nature of the cognitive studies performed limit the ability of accurately measuring such outcomes in rodents. More studies might be required to assess if BM-MSCs can improve cognitive function.

Based on the results of our work and prior meta-analyses (13–15), we believe that an overwhelming body of literature now supports the robust efficacy of BM-MSCs to improve functional outcomes. In fact, when we examined different time windows when MSC studies were completed over the past 18 years broken out into 2000–2008, 2009–2012, 2013–2015, and 2016–2018, we did not observe any differences in any of the four functional outcomes after BM-MSC therapy. These results show that there was likely conclusive evidence for the efficacy of BM-MSC many years ago. The data may also explain the negative correlation between year of publication of BM-MSC studies and impact factor, suggesting that redundant results showing functional improvement by BM-MSCs are being published in lower impact journals. The efficacy of BM-MSCs has also been shown across different countries and different laboratories. Animal protocols differ in different countries with their own review protocols and committees with different rules and regulations for animal surgeries. We found no differences in treatment effects across the globe except when using composite outcome scores where studies in Asia showed higher effect size as compared to studies in North America. To provide current up to date information that would be useful for the design of clinical trials, we performed a number of analyses on clinically relevant factors discussed below.

### Timing of BM-MSCs Administration

We found that overall effect-size for all outcome categories was consistently greatest when BM-MSCs were administered before 7 days. Within 7 days of stroke, we could not find consistent effects to determine a clear optimal therapeutic window. Administering BM-MSCs between 2 to 7 days leads to greatest benefit on composite score outcomes. On the other hand, administering BM-MSCs between 0 and 6 h led to the most significant improvement in sensorimotor outcomes. Although there are far less studies, BM-MSC administered after 7 days (compared with earlier time points) showed no improvement in composite scores, while MSCs still led to treatment effects, howbeit to a lesser extent, on motor and sensorimotor outcomes. Our results suggest that earlier administration of BM-MSCs before 7 days in rodents may be optimal to enhance functional recovery (25, 26) but MSCs may still confer less benefit when administered between 7 and 30 days in rodents, a time-period which many would consider a subacute to chronic period after stroke.

### Route of Administration

We found that intravenous administration resulted in significantly more improvement in motor function as compared to intracranial injection. Intravenous administration also showed a trend toward improving composite score and sensorimotor function, although this was not statistically significant. Intra-arterial administration showed similar trends of improving sensorimotor outcome as compared to intracranial administration. Our analyses do support that a systemic injection is preferable to intracranial injection within the early period after stroke, supporting the concept that MSCs act upon systemic responses after stroke. IV administration could direct MSCs to peripheral organs such as the lung and spleen (27–29), subsequently modulating release of trophic factors as well as immune responses from these organs (11, 12).

### Dose

When we analyzed dose of cells administered, we saw some trends for each category of outcomes (Figure S1). There was a trend of inverse relation between effectiveness of BM-MSCs and dose. On the other hand, there was a trend of direct correlation for improvement of sensorimotor function by BM-MSCs and dose. However, none of these trends were significant (Tables 2–4). We conclude that we could not find any clear dose response.

### Fresh vs. Cryopreserved BM-MSCs and Cell Passage

Various different passages, cryopreserved and fresh BM-MSCs exert functional benefits. Frozen BM-MSCs actually performed better than fresh BM-MSC in improving sensorimotor outcomes. These results do not support the belief that fresh cells are superior to frozen cells for studies involving focal ischemic stroke. Another surprising result was that BM-MSCs from passage 4 or higher produced significantly improved motor and sensorimotor outcome as compared to those from passage 2–4. However, we recommend to interpret these results with caution and recommend further detailed studies to analyze fresh vs. frozen and cell passage in future studies.

### Age

Age is an important factor for determining stroke functional outcomes. The aged brain has less regenerative potential and increased inflammatory responses to stroke (30–32). Even spontaneous recovery after stroke is delayed in aged animals (33, 34). In our meta-analysis, BM-MSC administration was tested in old animals in only 5 out of 141 articles. While our sample size does not provide adequate power to conduct reliable meta-regression to evaluate the effect of age, our analyses found that the pooled effect size was significant for composite score as well as sensorimotor function in both young and old stroke animals. However, the 95% confidence interval was wider for old animals due to less number of studies and more variability.

### Other Clinically Relevant Factors

Other factors that have not been studied well in prior meta-analyses were also examined. Labeling did not appear to alter the efficacy of BM-MSCs. These results support the use of labeled cells in clinical trials in order to track their migration and obtain needed data on biodistribution in patients. Not surprisingly, we found that animals without comorbidities improved significantly better after BM-MSCs treatment as compared to animals with co-morbidities. These results have implications for clinical trials and predicted effect sizes in patients with vascular risk factors and require further study. We hope that large clinical studies of BM-MSCs are funded that will permit sub hoc analyses to determine differential effects of vascular risk factors in patients treated with BM-MSCs. Lastly, we found intriguingly that female animals compared with males achieved better outcomes on sensorimotor outcomes after BM-MSC treatment but further studies will be needed to substantiate if there are true sex differences in the treatment effects of BM-MSCs.

## Conclusion

Given the wealth of preclinical data supporting the efficacy of BM-MSCs over 18 years, we recommend that this cellular therapy should be tested extensively in well-designed clinical trials in different time windows using different delivery routes. BM-MSCs may exert different treatment effects at various time points after stroke from early acute stages to later subacute and even chronic stroke. Clinical trials should pave the path forward for the further development of BM-MSCs in stroke patients.

## Author Contributions

NS wrote the manuscript. CC conducted the statistical analysis and wrote the statistical section in manuscript. NS, KG, DM, SG, KP, DN, KV, and JR collected the data. NS and KP designed the study. NS, FV, and SS oversaw the project and edited the manuscript.

### Conflict of Interest Statement

As an employee of the institution (UTHealth), SS has served in the following roles: as a site investigator in clinical trials sponsored by industry companies—Athersys, Genentech, Pfizer, Dart Neuroscience, and SanBio, for which UTHealth receives payments on the basis of clinical trial contracts; as an investigator on clinical trials supported by National Institutes of Health (NIH) grants, Department of Defense, Let's Cure CP, the Texas Institute for Rehabilitation and Research Foundation, and the Cord Blood Registry Systems; as a principal investigator on NIH-funded grants in basic science research; as principal investigator for an imaging analysis center for clinical trials sponsored by SanBio. Whereas, UTHealth uses SS with expertise in stroke, UTHealth has served as a consultant to Neuralstem, SanBio, Mesoblast, ReNeuron, Lumosa, Celgene, Dart Neuroscience, BlueRock and Aldagen. All funding goes to the institution. The remaining authors declare that the research was conducted in the absence of any commercial or financial relationships that could be construed as a potential conflict of interest.
