# Supplementary material for: World-Wide Efficacy of Bone Marrow Derived Mesenchymal Stromal Cells in Preclinical Ischemic Stroke Models: Systematic Review and Meta-Analysis
Source: Front Neurol. 2019 Apr 24;10:405. doi: 10.3389/fneur.2019.00405 (PMC6491581; doi:10.3389/fneur.2019.00405)
Supplement: Supplementary file 1 [file Data_Sheet_1.PDF]

World-Wide Efficacy of Bone Marrow Derived Mesenchymal Stromal Cells in Preclinical Ischemic Stroke Models: Systematic Review and Meta-Analysis

Supplementary Material

Figure S1. Scatterplot of effect size for functional outcome of all experiments in the meta-analysis plotted against dose of BM-MSCs (per kg) given to stroke animals. **A)** Scatterplot of effect size for composite scores vs. dose (cells/kg) showing downward trend with increasing dose (p=0.32); **B)** Scatterplot of effect size for motor function vs. dose (cells/kg) (p=0.83); **C)** Scatterplot of effect size for sensorimotor function vs. dose (cells/kg) showing upward trend with increasing dose (p=0.09); **D)** Scatterplot of effect size for cognitive function vs. dose (cells/kg) (p=0.99). The figure also shows 95% confidence intervals. None of these trends are statistically significant.

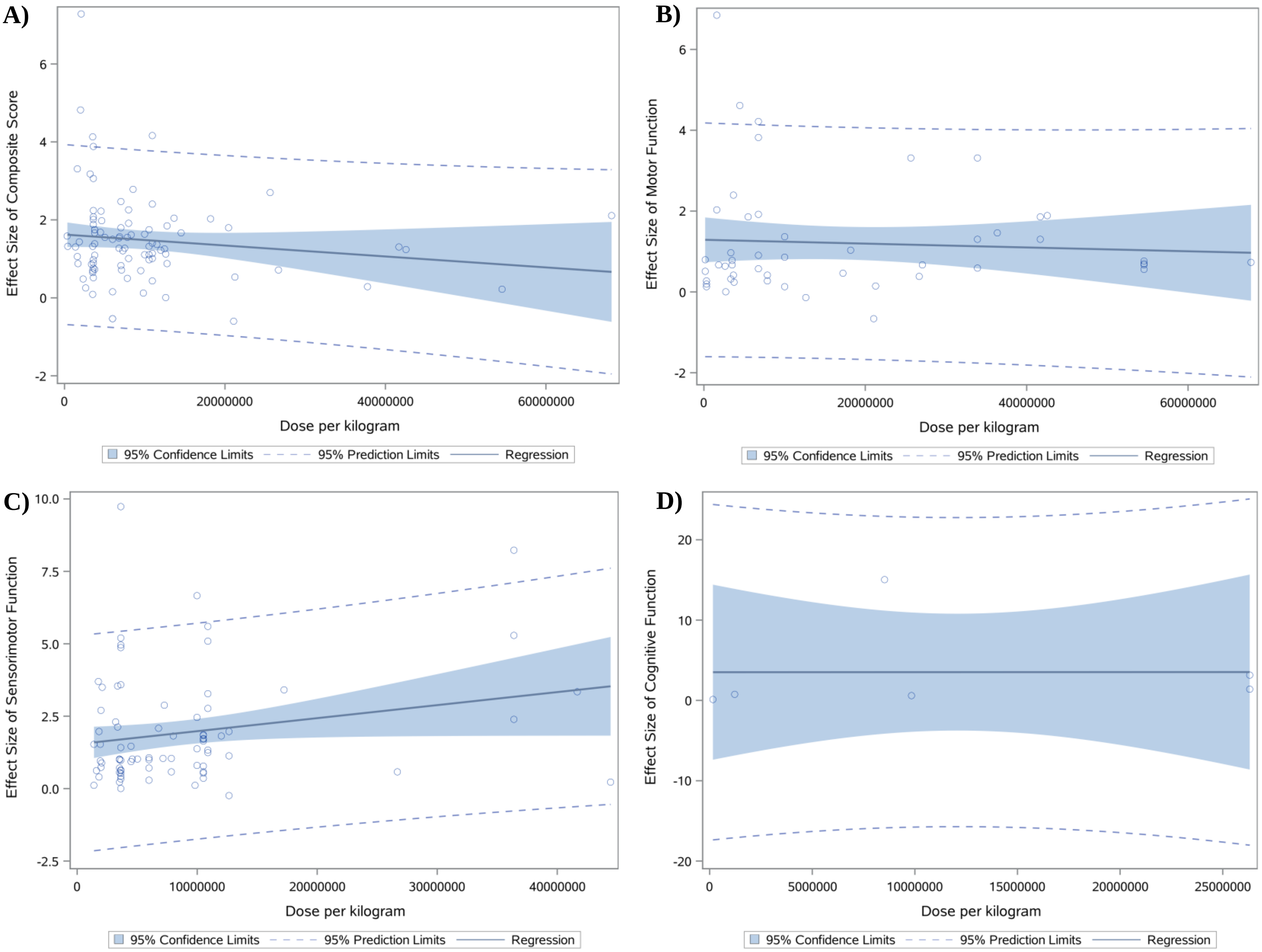

Figure S2. Forest Plot showing standardized mean difference of functional outcomes between BM-MSC therapy and control groups, when BM-MSCs were administered at or after 4 weeks of ischemic stroke. Cochran's Q-statistic revealed no heterogeneity among these six experiments (p=0.19). Overall treatment effect size was statistically significant (95% CI: 0.27 – 1.11).

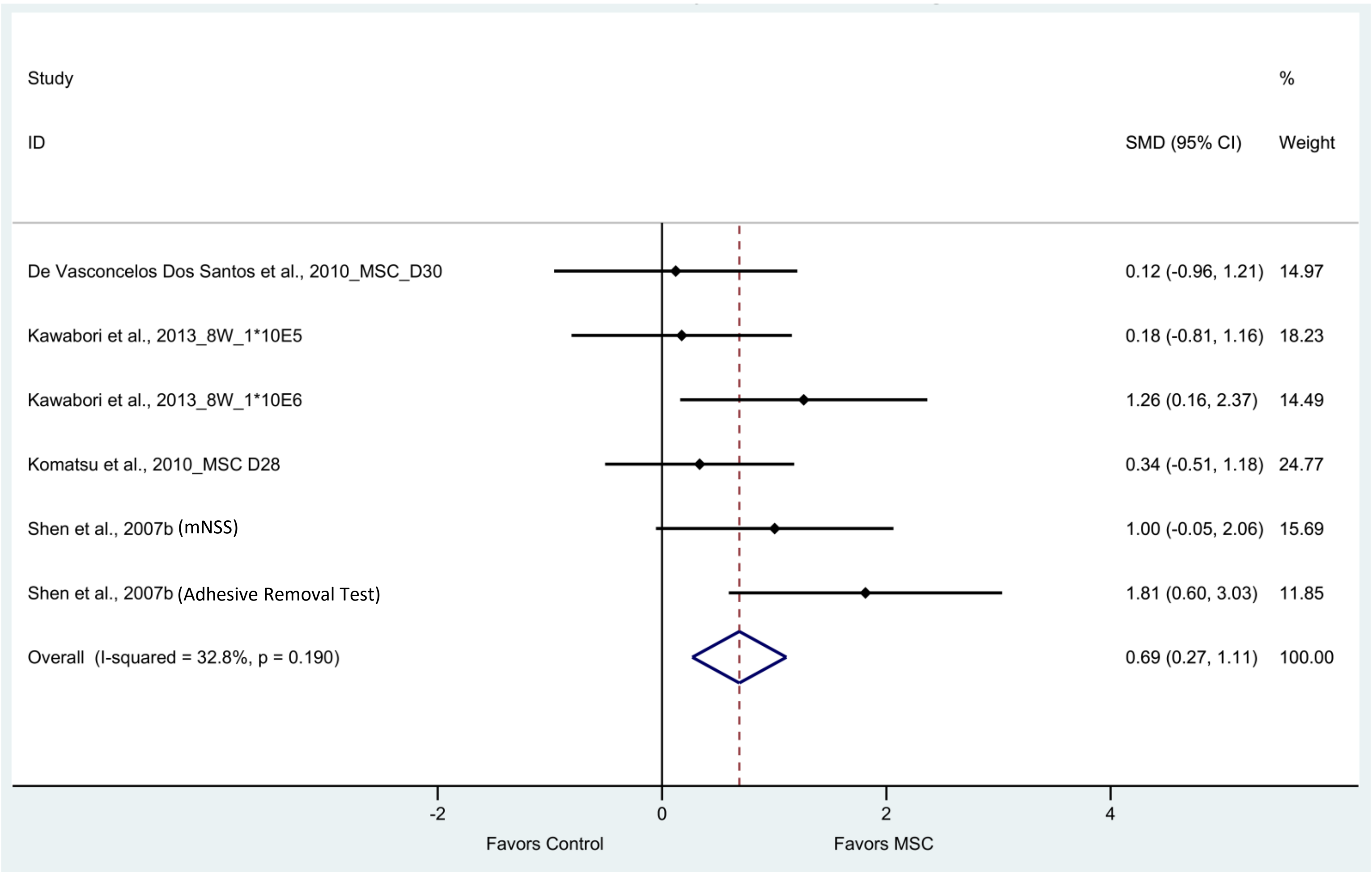

Table S1. Frequency and percentage of all interested variables by test categories.

Variable	Total	Composite Scores	Cognitive Function	Motor Function	Sensorimotor Function
**Cell Labeling,** n(%)	N=269	N=105	N=10	N=53	N=101
No	178 (66.2%)	66 (62.9%)	5 (50.0%)	39 (73.6%)	68 (67.3%)
Yes	91 (33.8%)	39 (37.1%)	5 (50.0%)	14 (26.4%)	33 (32.7%)
**Route of Administration,** n(%)	N=269	N=105	N=10	N=53	N=101
IA	24 (8.9%)	13 (12.4%)	0 (0.0%)	3 (5.7%)	8 (7.9%)
IC	75 (27.9%)	25 (23.8%)	5 (50.0%)	15 (28.3%)	30 (29.7%)
IV	167 (62.1%)	67 (63.8%)	5 (50.0%)	34 (64.2%)	61 (60.4%)
Intranasally	3 (1.1%)	0 (0.0%)	0 (0.0%)	1 (1.9%)	2 (2.0%)
**Time when outcome was measured,** n(%)	N=269	N=105	N=10	N=53	N=101
2-4 Weeks	73 (27.1%)	28 (26.7%)	2 (20.0%)	18 (34.0%)	25 (24.8%)
4-12 Weeks	65 (24.2%)	16 (15.2%)	5 (50.0%)	8 (15.1%)	36 (35.6%)
<2 Weeks	123 (45.7%)	58 (55.2%)	2 (20.0%)	26 (49.1%)	37 (36.6%)
>12 Weeks	8 (3.0%)	3 (2.9%)	1 (10.0%)	1 (1.9%)	3 (3.0%)
**Cell Dose,** n(%)	N=269	N=105	N=10	N=53	N=101
<=1*10E6	130 (48.3%)	42 (40.0%)	5 (50.0%)	28 (52.8%)	55 (54.5%)
>1*10E6	137 (50.9%)	62 (59.0%)	4 (40.0%)	25 (47.2%)	46 (45.5%)
Unknown	2 (0.7%)	1 (1.0%)	1 (10.0%)	0 (0.0%)	0 (0.0%)
**Timing of BM-MSC Administration,** n(%)	N=269	N=105	N=10	N=53	N=101
0-6 Hrs	60 (22.3%)	26 (24.8%)	1 (10.0%)	7 (13.2%)	26 (25.7%)
12-24 Hrs	124 (46.1%)	56 (53.3%)	3 (30.0%)	27 (50.9%)	38 (37.6%)
2-7 Days	68 (25.3%)	19 (18.1%)	5 (50.0%)	14 (26.4%)	30 (29.7%)
>7 Days	17 (6.3%)	4 (3.8%)	1 (10.0%)	5 (9.4%)	7 (6.9%)
**Fresh vs. Frozen BM-MSCs,** n(%)	N=269	N=105	N=10	N=53	N=101
Fresh	193 (71.7%)	82 (78.1%)	7 (70.0%)	39 (73.6%)	65 (64.4%)
Frozen	65 (24.2%)	21 (20.0%)	3 (30.0%)	12 (22.6%)	29 (28.7%)
Unknown	11 (4.1%)	2 (1.9%)	0 (0.0%)	2 (3.8%)	7 (6.9%)
**Passage of BM-MSCs,** n(%)	N=269	N=105	N=10	N=53	N=101
2-4	155 (57.6%)	68 (64.8%)	5 (50.0%)	29 (54.7%)	53 (52.5%)
>4	48 (17.8%)	16 (15.2%)	1 (10.0%)	13 (24.5%)	18 (17.8%)
Unknown	66 (24.5%)	21 (20.0%)	4 (40.0%)	11 (20.8%)	30 (29.7%)
**Species of BM-MSC donor,** n(%)	N=269	N=105	N=10	N=53	N=101
Dog MSC	3 (1.1%)	3 (2.9%)	0 (0.0%)	0 (0.0%)	0 (0.0%)
Human MSC	72 (26.8%)	24 (22.9%)	3 (30.0%)	12 (22.6%)	33 (32.7%)
Mouse MSC	15 (5.6%)	5 (4.8%)	1 (10.0%)	4 (7.5%)	5 (5.0%)
Rabbit MSC	1 (0.4%)	1 (1.0%)	0 (0.0%)	0 (0.0%)	0 (0.0%)

Variable	Total	Composite Scores	Cognitive Function	Motor Function	Sensorimotor Function
Rat MSC	178 (66.2%)	72 (68.6%)	6 (60.0%)	37 (69.8%)	63 (62.4%)
**Donor Gender,** n(%)	N=269	N=105	N=10	N=53	N=101
Female	12 (4.5%)	7 (6.7%)	1 (10.0%)	1 (1.9%)	3 (3.0%)
Male	83 (30.9%)	32 (30.5%)	4 (40.0%)	23 (43.4%)	24 (23.8%)
Unknown	174 (64.7%)	66 (62.9%)	5 (50.0%)	29 (54.7%)	74 (73.3%)
**Gender of Stroke Animal,** n(%)	N=269	N=105	N=10	N=53	N=101
Both	2 (0.7%)	2 (1.9%)	0 (0.0%)	0 (0.0%)	0 (0.0%)
Female	24 (8.9%)	9 (8.6%)	0 (0.0%)	1 (1.9%)	14 (13.9%)
Male	221 (82.2%)	83 (79.0%)	10 (100.0%)	49 (92.5%)	79 (78.2%)
Unknown	22 (8.2%)	11 (10.5%)	0 (0.0%)	3 (5.7%)	8 (7.9%)
**Species of Stroke Animal,** n(%)	N=269	N=105	N=10	N=53	N=101
Dog	3 (1.1%)	3 (2.9%)	0 (0.0%)	0 (0.0%)	0 (0.0%)
Mice	27 (10.0%)	8 (7.6%)	2 (20.0%)	6 (11.3%)	11 (10.9%)
Monkey	1 (0.4%)	1 (1.0%)	0 (0.0%)	0 (0.0%)	0 (0.0%)
Rabbit	1 (0.4%)	1 (1.0%)	0 (0.0%)	0 (0.0%)	0 (0.0%)
Rat	237 (88.1%)	92 (87.6%)	8 (80.0%)	47 (88.7%)	90 (89.1%)
**Age of Stroke Animal,** n(%)	N=269	N=105	N=10	N=53	N=101
Adult	254 (94.4%)	97 (92.4%)	10 (100.0%)	52 (98.1%)	95 (94.1%)
Neonate	2 (0.7%)	0 (0.0%)	0 (0.0%)	1 (1.9%)	1 (1.0%)
Retired Breeder	10 (3.7%)	5 (4.8%)	0 (0.0%)	0 (0.0%)	5 (5.0%)
Unknown	3 (1.1%)	3 (2.9%)	0 (0.0%)	0 (0.0%)	0 (0.0%)
**Co-morbidities,** n(%)	N=269	N=105	N=10	N=53	N=101
Normal	255 (94.8%)	100 (95.2%)	9 (90.0%)	48 (90.6%)	98 (97.0%)
SCID	2 (0.7%)	0 (0.0%)	1 (10.0%)	0 (0.0%)	1 (1.0%)
T1-DM	7 (2.6%)	3 (2.9%)	0 (0.0%)	3 (5.7%)	1 (1.0%)
T2-DM	5 (1.9%)	2 (1.9%)	0 (0.0%)	2 (3.8%)	1 (1.0%)
**Continent,** n(%)	N=269	N=105	N=10	N=53	N=101
Asia	151 (56.1%)	65 (61.9%)	6 (60.0%)	21 (39.6%)	59 (58.4%)
Europe	23 (8.6%)	9 (8.6%)	4 (40.0%)	4 (7.5%)	6 (5.9%)
North America	91 (33.8%)	31 (29.5%)	0 (0.0%)	25 (47.2%)	35 (34.7%)
South America	4 (1.5%)	0 (0.0%)	0 (0.0%)	3 (5.7%)	1 (1.0%)
**Country,** n(%)	N=269	N=105	N=10	N=53	N=101
Brazil	4 (1.5%)	0 (0.0%)	0 (0.0%)	3 (5.7%)	1 (1.0%)
Canada	4 (1.5%)	3 (2.9%)	0 (0.0%)	0 (0.0%)	1 (1.0%)
China	68 (25.3%)	44 (41.9%)	2 (20.0%)	9 (17.0%)	13 (12.9%)
Finland	2 (0.7%)	0 (0.0%)	0 (0.0%)	2 (3.8%)	0 (0.0%)
France	3 (1.1%)	1 (1.0%)	1 (10.0%)	0 (0.0%)	1 (1.0%)

Variable	Total	Composite Scores	Cognitive Function	Motor Function	Sensorimotor Function
Germany	3 (1.1%)	0 (0.0%)	0 (0.0%)	1 (1.9%)	2 (2.0%)
Iran	6 (2.2%)	3 (2.9%)	0 (0.0%)	2 (3.8%)	1 (1.0%)
Italy	7 (2.6%)	5 (4.8%)	1 (10.0%)	0 (0.0%)	1 (1.0%)
Japan	53 (19.7%)	10 (9.5%)	4 (40.0%)	7 (13.2%)	32 (31.7%)
Netherlands	2 (0.7%)	0 (0.0%)	0 (0.0%)	1 (1.9%)	1 (1.0%)
Russia	2 (0.7%)	0 (0.0%)	2 (20.0%)	0 (0.0%)	0 (0.0%)
South Korea	23 (8.6%)	8 (7.6%)	0 (0.0%)	3 (5.7%)	12 (11.9%)
Spain	4 (1.5%)	3 (2.9%)	0 (0.0%)	0 (0.0%)	1 (1.0%)
Taiwan	1 (0.4%)	0 (0.0%)	0 (0.0%)	0 (0.0%)	1 (1.0%)
USA	87 (32.3%)	28 (26.7%)	0 (0.0%)	25 (47.2%)	34 (33.7%)
**Institute,** n(%)	N=269	N=105	N=10	N=53	N=101
Capital Medical University	2 (0.7%)	0 (0.0%)	0 (0.0%)	2 (3.8%)	0 (0.0%)
Centro de Biociências e Biotecnologia	2 (0.7%)	0 (0.0%)	0 (0.0%)	1 (1.9%)	1 (1.0%)
Cidade Universitária- Ilha do Fundão	2 (0.7%)	0 (0.0%)	0 (0.0%)	2 (3.8%)	0 (0.0%)
Emory University School of Medicine	3 (1.1%)	0 (0.0%)	0 (0.0%)	0 (0.0%)	3 (3.0%)
Federico II University Medical School	3 (1.1%)	3 (2.9%)	0 (0.0%)	0 (0.0%)	0 (0.0%)
Fujian Medical University	2 (0.7%)	2 (1.9%)	0 (0.0%)	0 (0.0%)	0 (0.0%)
General Hospital of Chinese PLA	2 (0.7%)	1 (1.0%)	0 (0.0%)	0 (0.0%)	1 (1.0%)
General Hospital of Tianjin Medical University	1 (0.4%)	1 (1.0%)	0 (0.0%)	0 (0.0%)	0 (0.0%)
Guangdong Provincial Hospital of Chinese Medicine	1 (0.4%)	1 (1.0%)	0 (0.0%)	0 (0.0%)	0 (0.0%)
Guangxi Medical University	1 (0.4%)	1 (1.0%)	0 (0.0%)	0 (0.0%)	0 (0.0%)
Hanyang University	1 (0.4%)	0 (0.0%)	0 (0.0%)	0 (0.0%)	1 (1.0%)
Harbin Medical University	1 (0.4%)	1 (1.0%)	0 (0.0%)	0 (0.0%)	0 (0.0%)
Henry Ford Health Science Center	61 (22.7%)	24 (22.9%)	0 (0.0%)	9 (17.0%)	28 (27.7%)
Hiroshima University	2 (0.7%)	1 (1.0%)	0 (0.0%)	0 (0.0%)	1 (1.0%)
Hokkaido University Graduate School of Medicine	11 (4.1%)	1 (1.0%)	1 (10.0%)	0 (0.0%)	9 (8.9%)
Hospital La Paz/Autonoma University School of Medicine	2 (0.7%)	2 (1.9%)	0 (0.0%)	0 (0.0%)	0 (0.0%)
IRCCS – Istituto di Ricerche Farmacologiche Mario Negri	4 (1.5%)	2 (1.9%)	1 (10.0%)	0 (0.0%)	1 (1.0%)
Iran University of Medical Sciences	2 (0.7%)	1 (1.0%)	0 (0.0%)	1 (1.9%)	0 (0.0%)
Jiangxi Provincial People’s Hospital	6 (2.2%)	3 (2.9%)	0 (0.0%)	0 (0.0%)	3 (3.0%)
Kyoto University	2 (0.7%)	0 (0.0%)	1 (10.0%)	1 (1.9%)	0 (0.0%)
Kyung Hee University	3 (1.1%)	1 (1.0%)	0 (0.0%)	0 (0.0%)	2 (2.0%)
Liaoning Medical University	1 (0.4%)	1 (1.0%)	0 (0.0%)	0 (0.0%)	0 (0.0%)
Loyola University Chicago	1 (0.4%)	0 (0.0%)	0 (0.0%)	1 (1.9%)	0 (0.0%)

Variable	Total	Composite Scores	Cognitive Function	Motor Function	Sensorimotor Function
Macau University of Science and Technology	3 (1.1%)	3 (2.9%)	0 (0.0%)	0 (0.0%)	0 (0.0%)
Nagasaki University	2 (0.7%)	1 (1.0%)	0 (0.0%)	1 (1.9%)	0 (0.0%)
Nanjing Medical University	4 (1.5%)	3 (2.9%)	0 (0.0%)	1 (1.9%)	0 (0.0%)
Nantong University	3 (1.1%)	1 (1.0%)	0 (0.0%)	1 (1.9%)	1 (1.0%)
National Chung-Hsing University	1 (0.4%)	0 (0.0%)	0 (0.0%)	0 (0.0%)	1 (1.0%)
Neuroscience and Cerebrovascular Research Laboratory	2 (0.7%)	1 (1.0%)	0 (0.0%)	0 (0.0%)	1 (1.0%)
Nippon Medical School	2 (0.7%)	2 (1.9%)	0 (0.0%)	0 (0.0%)	0 (0.0%)
No.148 Hospital of PLA	1 (0.4%)	1 (1.0%)	0 (0.0%)	0 (0.0%)	0 (0.0%)
Osaka Medical College	4 (1.5%)	4 (3.8%)	0 (0.0%)	0 (0.0%)	0 (0.0%)
Peking Union Medical College	5 (1.9%)	3 (2.9%)	0 (0.0%)	0 (0.0%)	2 (2.0%)
Sapporo Medical University School of Medicine	20 (7.4%)	1 (1.0%)	0 (0.0%)	3 (5.7%)	16 (15.8%)
Second Affiliated Hospital of Harbin Medical University	1 (0.4%)	1 (1.0%)	0 (0.0%)	0 (0.0%)	0 (0.0%)
Semnan University of Medical Sciences	2 (0.7%)	1 (1.0%)	0 (0.0%)	1 (1.9%)	0 (0.0%)
Seoul National University	1 (0.4%)	1 (1.0%)	0 (0.0%)	0 (0.0%)	0 (0.0%)
Shandong University School of Medicine	1 (0.4%)	1 (1.0%)	0 (0.0%)	0 (0.0%)	0 (0.0%)
Shiraz University of Medical Sciences	1 (0.4%)	1 (1.0%)	0 (0.0%)	0 (0.0%)	0 (0.0%)
Sichuan University	2 (0.7%)	2 (1.9%)	0 (0.0%)	0 (0.0%)	0 (0.0%)
Southern Medical University	1 (0.4%)	1 (1.0%)	0 (0.0%)	0 (0.0%)	0 (0.0%)
Sun Yat-Sen University	3 (1.1%)	2 (1.9%)	1 (10.0%)	0 (0.0%)	0 (0.0%)
Tehran University of Medical Sciences	1 (0.4%)	0 (0.0%)	0 (0.0%)	0 (0.0%)	1 (1.0%)
The Affiliated Hospital of Xuzhou Medical College	2 (0.7%)	1 (1.0%)	0 (0.0%)	1 (1.9%)	0 (0.0%)
The BenQ Neurological Institute of Nanjing Medical University	2 (0.7%)	0 (0.0%)	0 (0.0%)	1 (1.9%)	1 (1.0%)
The Catholic University of Korea	6 (2.2%)	1 (1.0%)	0 (0.0%)	0 (0.0%)	5 (5.0%)
The First Affiliated Hospital of Harbin Medical University	2 (0.7%)	0 (0.0%)	0 (0.0%)	1 (1.9%)	1 (1.0%)
The First Affiliated Hospital of Nanjing Medical University	3 (1.1%)	3 (2.9%)	0 (0.0%)	0 (0.0%)	0 (0.0%)
The First Affiliated Hospital of Xiamen University	1 (0.4%)	1 (1.0%)	0 (0.0%)	0 (0.0%)	0 (0.0%)
The First Bethune Hospital of Jilin University	1 (0.4%)	1 (1.0%)	0 (0.0%)	0 (0.0%)	0 (0.0%)
The Second Hospital of Tianjin Medical University	1 (0.4%)	1 (1.0%)	0 (0.0%)	0 (0.0%)	0 (0.0%)
The Third Affiliated Hospital of Xi'an Jiaotong University; Shaanxi Provincial	1 (0.4%)	1 (1.0%)	0 (0.0%)	0 (0.0%)	0 (0.0%)

Variable	Total	Composite Scores	Cognitive Function	Motor Function	Sensorimotor Function
The first affiliated hospital of Jinan University	1 (0.4%)	0 (0.0%)	0 (0.0%)	1 (1.9%)	0 (0.0%)
Third Military Medical University	1 (0.4%)	0 (0.0%)	1 (10.0%)	0 (0.0%)	0 (0.0%)
Thomas Jefferson University	5 (1.9%)	2 (1.9%)	0 (0.0%)	2 (3.8%)	1 (1.0%)
Tianjin General Hospital of Tianjin Medical University	3 (1.1%)	1 (1.0%)	0 (0.0%)	1 (1.9%)	1 (1.0%)
Toronto Western Research Institute	2 (0.7%)	1 (1.0%)	0 (0.0%)	0 (0.0%)	1 (1.0%)
Trans-Technologies, Ltd	2 (0.7%)	0 (0.0%)	2 (20.0%)	0 (0.0%)	0 (0.0%)
Union Hospital, Fujian Medical University	1 (0.4%)	1 (1.0%)	0 (0.0%)	0 (0.0%)	0 (0.0%)
Unité de Thérapie et d'Ingénierie Cellulaire	3 (1.1%)	1 (1.0%)	1 (10.0%)	0 (0.0%)	1 (1.0%)
University Hospital Essen, University of Duisburg-Essen	3 (1.1%)	0 (0.0%)	0 (0.0%)	1 (1.9%)	2 (2.0%)
University Medical Center	2 (0.7%)	0 (0.0%)	0 (0.0%)	1 (1.9%)	1 (1.0%)
University of Eastern Finland	2 (0.7%)	0 (0.0%)	0 (0.0%)	2 (3.8%)	0 (0.0%)
University of North Texas Health Science Center	14 (5.2%)	1 (1.0%)	0 (0.0%)	13 (24.5%)	0 (0.0%)
University of Toronto	2 (0.7%)	2 (1.9%)	0 (0.0%)	0 (0.0%)	0 (0.0%)
University of Toyama	7 (2.6%)	0 (0.0%)	1 (10.0%)	0 (0.0%)	6 (5.9%)
Weil Institute of Critical Care Medicine	3 (1.1%)	1 (1.0%)	0 (0.0%)	0 (0.0%)	2 (2.0%)
Xuzhou Medical University	2 (0.7%)	2 (1.9%)	0 (0.0%)	0 (0.0%)	0 (0.0%)
Yokohama City University Graduate School of Medicine	3 (1.1%)	0 (0.0%)	1 (10.0%)	2 (3.8%)	0 (0.0%)
Yonsei University Health System	12 (4.5%)	5 (4.8%)	0 (0.0%)	3 (5.7%)	4 (4.0%)
Zhejiang Chinese Medical University	3 (1.1%)	1 (1.0%)	0 (0.0%)	0 (0.0%)	2 (2.0%)
Zhujiang Hospital, Southern Medical University	3 (1.1%)	2 (1.9%)	0 (0.0%)	0 (0.0%)	1 (1.0%)
**Year Group,** n(%)	N=269	N=105	N=10	N=53	N=101
2000-2008	75 (27.9%)	25 (23.8%)	3 (30.0%)	6 (11.3%)	41 (40.6%)
2009-2012	62 (23.0%)	25 (23.8%)	3 (30.0%)	11 (20.8%)	23 (22.8%)
2013-2015	75 (27.9%)	26 (24.8%)	2 (20.0%)	22 (41.5%)	25 (24.8%)
2016-2018	57 (21.2%)	29 (27.6%)	2 (20.0%)	14 (26.4%)	12 (11.9%)

Table S2. Table showing pooled effect size for composite score and sensorimotor function between BM-MSC therapy and control groups, stratified by age of stroke animals. 95% confidence intervals are shown. p-values show significant effect size for BM-MSC treated group as compared to control group. Only 5 studies measured outcomes in old animals.

Functional Outcome vs. Age	Pooled Effect Size	95% Confidence Interval	p-value
**Composite Score**			
Adult	1.257	1.089 – 1.425	**< 0.0001**
Old (Retired Breeder)	1.499	1.071 – 1.927	**< 0.0001**
**Sensorimotor Function**			
Adult	1.367	1.151 – 1.583	**< 0.0001**
Old (Retired Breeder)	1.364	0.846 – 1.883	**< 0.0001**

REFERENCES FOR ARTICLES SELECTED FOR META-ANALYSIS

1. Akhoundzadeh K, Vakili A, Sameni HR, Vafaei AA, Rashidy-Pour A, Safari M, et al. Effects of the combined treatment of bone marrow stromal cells with mild exercise and thyroid hormone on brain damage and apoptosis in a mouse focal cerebral ischemia model. Metabolic brain disease. 2017;32(4):1267-77.
2. Andrews EM, Tsai SY, Johnson SC, Farrer JR, Wagner JP, Kopen GC, et al. Human adult bone marrow-derived somatic cell therapy results in functional recovery and axonal plasticity following stroke in the rat. Exp Neurol. 2008;211(2):588-92.
3. Bao C, Wang Y, Min H, Zhang M, Du X, Han R, et al. Combination of ginsenoside Rg1 and bone marrow mesenchymal stem cell transplantation in the treatment of cerebral ischemia reperfusion injury in rats. Cellular physiology and biochemistry : international journal of experimental cellular physiology, biochemistry, and pharmacology. 2015;37(3):901-10.
4. Bao X, Feng M, Wei J, Han Q, Zhao H, Li G, et al. Transplantation of Flk-1+ human bone marrow-derived mesenchymal stem cells promotes angiogenesis and neurogenesis after cerebral ischemia in rats. Eur J Neurosci. 2011;34(1):87-98.
5. Bao X, Wei J, Feng M, Lu S, Li G, Dou W, et al. Transplantation of human bone marrow-derived mesenchymal stem cells promotes behavioral recovery and endogenous neurogenesis after cerebral ischemia in rats. Brain Res. 2011;1367:103-13.
6. Bi M, Wang J, Zhang Y, Li L, Wang L, Yao R, et al. Bone mesenchymal stem cells transplantation combined with mild hypothermia improves the prognosis of cerebral ischemia in rats. PloS one. 2018;13(8):e0197405.
7. Cai K, Di Q, Shi J, Zhang Y. Dynamic changes of cell cycle elements in the ischemic brain after bone marrow stromal cells transplantation in rats. Neurosci Lett. 2009;467(1):15-9.
8. Chen C, Cheng Y, Chen J. Transfection of Noggin in bone marrow stromal cells (BMSCs) enhances BMSC-induced functional outcome after stroke in rats. J Neurosci Res. 2011;89(8):1194-202.
9. Chen J, Li Y, Katakowski M, Chen X, Wang L, Lu D, et al. Intravenous bone marrow stromal cell therapy reduces apoptosis and promotes endogenous cell proliferation after stroke in female rat. J Neurosci Res. 2003;73(6):778-86.
10. Chen J, Li Y, Wang L, Lu M, Chopp M. Caspase inhibition by Z-VAD increases the survival of grafted bone marrow cells and improves functional outcome after MCAo in rats. J Neurol Sci. 2002;199(1-2):17-24.
11. Chen J, Li Y, Wang L, Lu M, Zhang X, Chopp M. Therapeutic benefit of intracerebral transplantation of bone marrow stromal cells after cerebral ischemia in rats. J Neurol Sci. 2001;189(1-2):49-57.
12. Chen J, Li Y, Wang L, Zhang Z, Lu D, Lu M, et al. Therapeutic benefit of intravenous administration of bone marrow stromal cells after cerebral ischemia in rats. Stroke. 2001;32(4):1005-11.
13. Chen J, Li Y, Zhang R, Katakowski M, Gautam SC, Xu Y, et al. Combination therapy of stroke in rats with a nitric oxide donor and human bone marrow stromal cells enhances angiogenesis and neurogenesis. Brain Res. 2004;1005(1-2):21-8.
14. Chen J, Yang Y, Shen L, Ding W, Chen X, Wu E, et al. Hypoxic Preconditioning Augments the Therapeutic Efficacy of Bone Marrow Stromal Cells in a Rat Ischemic Stroke Model. Cellular and molecular neurobiology. 2017;37(6):1115-29.
15. Chen J, Ye X, Yan T, Zhang C, Yang XP, Cui X, et al. Adverse effects of bone marrow stromal cell treatment of stroke in diabetic rats. Stroke. 2011;42(12):3551-8.
16. Chen JR, Cheng GY, Sheu CC, Tseng GF, Wang TJ, Huang YS. Transplanted bone marrow stromal cells migrate, differentiate and improve motor function in rats with experimentally induced cerebral stroke. J Anat. 2008;213(3):249-58.

17. Chen ZZ, Jiang XD, Zhang LL, Shang JH, Du MX, Xu G, et al. Beneficial effect of autologous transplantation of bone marrow stromal cells and endothelial progenitor cells on cerebral ischemia in rabbits. Neurosci Lett. 2008;445(1):36-41.
18. Cho DY, Jeun SS. Combination therapy of human bone marrow-derived mesenchymal stem cells and minocycline improves neuronal function in a rat middle cerebral artery occlusion model. Stem Cell Res Ther. 2018;9(1):309.
19. Cho GW, Koh SH, Kim MH, Yoo AR, Noh MY, Oh S, et al. The neuroprotective effect of erythropoietin-transduced human mesenchymal stromal cells in an animal model of ischemic stroke. Brain Res. 2010;1353:1-13.
20. Cui C, Ye X, Chopp M, Venkat P, Zacharek A, Yan T, et al. miR-145 Regulates Diabetes-Bone Marrow Stromal Cell-Induced Neurorestorative Effects in Diabetes Stroke Rats. Stem cells translational medicine. 2016;5(12):1656-67.
21. Cui X, Chen J, Zacharek A, Li Y, Roberts C, Kapke A, et al. Nitric oxide donor upregulation of stromal cell-derived factor-1/chemokine (CXC motif) receptor 4 enhances bone marrow stromal cell migration into ischemic brain after stroke. Stem Cells. 2007;25(11):2777-85.
22. Cui X, Chopp M, Zacharek A, Roberts C, Lu M, Savant-Bhonsale S, et al. Chemokine, vascular and therapeutic effects of combination Simvastatin and BMSC treatment of stroke. Neurobiol Dis. 2009;36(1):35-41.
23. de Freitas HT, da Silva VG, Giraldi-Guimaraes A. Comparative study between bone marrow mononuclear fraction and mesenchymal stem cells treatment in sensorimotor recovery after focal cortical ablation in rats. Behavioral and brain functions : BBF. 2012;8:58.
24. de Vasconcelos Dos Santos A, da Costa Reis J, Diaz Paredes B, Moraes L, Jasmin, Giraldi-Guimaraes A, et al. Therapeutic window for treatment of cortical ischemia with bone marrow-derived cells in rats. Brain Res. 2010;1306:149-58.
25. Deng YB, Ye WB, Hu ZZ, Yan Y, Wang Y, Takon BF, et al. Intravenously administered BMSCs reduce neuronal apoptosis and promote neuronal proliferation through the release of VEGF after stroke in rats. Neurological research. 2010;32(2):148-56.
26. Ding J, Cheng Y, Gao S, Chen J. Effects of nerve growth factor and Noggin-modified bone marrow stromal cells on stroke in rats. J Neurosci Res. 2011;89(2):222-30.
27. Ding X, Li Y, Liu Z, Zhang J, Cui Y, Chen X, et al. The sonic hedgehog pathway mediates brain plasticity and subsequent functional recovery after bone marrow stromal cell treatment of stroke in mice. J Cereb Blood Flow Metab. 2013;33(7):1015-24.
28. Doeppner TR, Herz J, Gorgens A, Schlechter J, Ludwig AK, Radtke S, et al. Extracellular Vesicles Improve Post-Stroke Neuroregeneration and Prevent Postischemic Immunosuppression. Stem cells translational medicine. 2015;4(10):1131-43.
29. Du S, Guan J, Mao G, Liu Y, Ma S, Bao X, et al. Intra-arterial delivery of human bone marrow mesenchymal stem cells is a safe and effective way to treat cerebral ischemia in rats. Cell transplantation. 2014;23 Suppl 1:S73-82.
30. Feng N, Hao G, Yang F, Qu F, Zheng H, Liang S, et al. Transplantation of mesenchymal stem cells promotes the functional recovery of the central nervous system following cerebral ischemia by inhibiting myelin-associated inhibitor expression and neural apoptosis. Experimental and therapeutic medicine. 2016;11(5):1595-600.
31. Fukuda Y, Horie N, Satoh K, Yamaguchi S, Morofuji Y, Hiu T, et al. Intra-arterial transplantation of low-dose stem cells provides functional recovery without adverse effects after stroke. Cellular and molecular neurobiology. 2015;35(3):399-406.
32. Goldmacher GV, Nasser R, Lee DY, Yigit S, Rosenwasser R, Iacovitti L. Tracking transplanted bone marrow stem cells and their effects in the rat MCAO stroke model. PloS one. 2013;8(3):e60049.

33. Gong Z, Ran H, Wu S, Zhu J, Zheng J. Ultrasound-microbubble transplantation of bone marrow stromal cells improves neurological function after forebrain ischemia in adult mice. Cell Biochem Biophys. 2014;70(1):499-504.
34. Guo JW, Chen C, Huang Y, Li B. Combinatorial effects of naomai yihao capsules and vascular endothelial growth factor gene-transfected bone marrow mesenchymal stem cells on angiogenesis in cerebral ischemic tissues in rats. J Tradit Chin Med. 2012;32(1):87-92.
35. Gutierrez-Fernandez M, Rodriguez-Frutos B, Alvarez-Grech J, Vallejo-Cremades MT, Exposito-Alcaide M, Merino J, et al. Functional recovery after hematic administration of allogenic mesenchymal stem cells in acute ischemic stroke in rats. Neuroscience. 2011;175:394-405.
36. Gutierrez-Fernandez M, Rodriguez-Frutos B, Ramos-Cejudo J, Teresa Vallejo-Cremades M, Fuentes B, Cerdan S, et al. Effects of intravenous administration of allogenic bone marrow- and adipose tissue-derived mesenchymal stem cells on functional recovery and brain repair markers in experimental ischemic stroke. Stem Cell Res Ther. 2013;4(1):11.
37. Hayase M, Kitada M, Wakao S, Itokazu Y, Nozaki K, Hashimoto N, et al. Committed neural progenitor cells derived from genetically modified bone marrow stromal cells ameliorate deficits in a rat model of stroke. J Cereb Blood Flow Metab. 2009;29(8):1409-20.
38. He H, Zeng Q, Huang G, Lin Y, Lin H, Liu W, et al. Bone marrow mesenchymal stem cell transplantation exerts neuroprotective effects following cerebral ischemia/reperfusion injury by inhibiting autophagy via the PI3K/Akt pathway. Brain Res. 2018.
39. He X, Jiang L, Dan QQ, Lv Q, Hu Y, Liu J, et al. Bone marrow stromal cells promote neuroplasticity of cerebral ischemic rats via a phosphorylated CRMP2-mediated mechanism. Behavioural brain research. 2017;320:494-503.
40. He XY, Chen ZZ, Cai YQ, Xu G, Shang JH, Kou SB, et al. Expression of cytokines in rat brain with focal cerebral ischemia after grafting with bone marrow stromal cells and endothelial progenitor cells. Cytotherapy. 2011;13(1):46-53.
41. Heo JS, Choi SM, Kim HO, Kim EH, You J, Park T, et al. Neural transdifferentiation of human bone marrow mesenchymal stem cells on hydrophobic polymer-modified surface and therapeutic effects in an animal model of ischemic stroke. Neuroscience. 2013;238:305-18.
42. Hokari M, Kuroda S, Chiba Y, Maruichi K, Iwasaki Y. Synergistic effects of granulocyte-colony stimulating factor on bone marrow stromal cell transplantation for mice cerebral infarct. Cytokine. 2009;46(2):260-6.
43. Horita Y, Honmou O, Harada K, Houkin K, Hamada H, Kocsis JD. Intravenous administration of glial cell line-derived neurotrophic factor gene-modified human mesenchymal stem cells protects against injury in a cerebral ischemia model in the adult rat. J Neurosci Res. 2006;84(7):1495-504.
44. Hosseini SM, Farahmandnia M, Razi Z, Delavari S, Shakibajahromi B, Sarvestani FS, et al. Combination cell therapy with mesenchymal stem cells and neural stem cells for brain stroke in rats. International journal of stem cells. 2015;8(1):99-105.
45. Hu J, Liu B, Zhao Q, Jin P, Hua F, Zhang Z, et al. Bone marrow stromal cells inhibits HMGB1-mediated inflammation after stroke in type 2 diabetic rats. Neuroscience. 2016;324:11-9.
46. Hu Y, Xiong LL, Zhang P, Wang TH. Microarray expression profiles of genes in lung tissues of rats subjected to focal cerebral ischemia-induced lung injury following bone marrow-derived mesenchymal stem cell transplantation. International journal of molecular medicine. 2017;39(1):57-70.
47. Huang W, Mo X, Qin C, Zheng J, Liang Z, Zhang C. Transplantation of differentiated bone marrow stromal cells promotes motor functional recovery in rats with stroke. Neurological research. 2013;35(3):320-8.
48. Ikeda N, Nonoguchi N, Zhao MZ, Watanabe T, Kajimoto Y, Furutama D, et al. Bone marrow stromal cells that enhanced fibroblast growth factor-2 secretion by herpes simplex virus vector improve neurological outcome after transient focal cerebral ischemia in rats. Stroke. 2005;36(12):2725-30.

49. Jang DK, Park SI, Han YM, Jang KS, Park MS, Chung YA, et al. Motor-evoked potential confirmation of functional improvement by transplanted bone marrow mesenchymal stem cell in the ischemic rat brain. J Biomed Biotechnol. 2011;2011:238409.
50. Jeong CH, Kim SM, Lim JY, Ryu CH, Jun JA, Jeun SS. Mesenchymal stem cells expressing brain-derived neurotrophic factor enhance endogenous neurogenesis in an ischemic stroke model. Biomed Res Int. 2014;2014:129145.
51. Kawabori M, Kuroda S, Ito M, Shichinohe H, Houkin K, Kuge Y, et al. Timing and cell dose determine therapeutic effects of bone marrow stromal cell transplantation in rat model of cerebral infarct. Neuropathology. 2013;33(2):140-8.
52. Kawabori M, Kuroda S, Sugiyama T, Ito M, Shichinohe H, Houkin K, et al. Intracerebral, but not intravenous, transplantation of bone marrow stromal cells enhances functional recovery in rat cerebral infarct: an optical imaging study. Neuropathology. 2012;32(3):217-26.
53. Komatsu K, Honmou O, Suzuki J, Houkin K, Hamada H, Kocsis JD. Therapeutic time window of mesenchymal stem cells derived from bone marrow after cerebral ischemia. Brain Res. 2010;1334:84-92.
54. Kurozumi K, Nakamura K, Tamiya T, Kawano Y, Kobune M, Hirai S, et al. BDNF gene-modified mesenchymal stem cells promote functional recovery and reduce infarct size in the rat middle cerebral artery occlusion model. Mol Ther. 2004;9(2):189-97.
55. Lapi D, Vagnani S, Sapio D, Mastantuono T, Boscia F, Pignataro G, et al. Effects of bone marrow mesenchymal stem cells (BM-MSCs) on rat pial microvascular remodeling after transient middle cerebral artery occlusion. Front Cell Neurosci. 2015;9:329.
56. Li L, Chu L, Fang Y, Yang Y, Qu T, Zhang J, et al. Preconditioning of bone marrow-derived mesenchymal stromal cells by tetramethylpyrazine enhances cell migration and improves functional recovery after focal cerebral ischemia in rats. Stem Cell Res Ther. 2017;8(1):112.
57. Li N, Wang P, Ma XL, Wang J, Zhao LJ, Du L, et al. Effect of bone marrow stromal cell transplantation on neurologic function and expression of VEGF in rats with focal cerebral ischemia. Molecular medicine reports. 2014;10(5):2299-305.
58. Li X, Huang M, Zhao R, Zhao C, Liu Y, Zou H, et al. Intravenously Delivered Allogeneic Mesenchymal Stem Cells Bidirectionally Regulate Inflammation and Induce Neurotrophic Effects in Distal Middle Cerebral Artery Occlusion Rats Within the First 7 Days After Stroke. Cellular physiology and biochemistry : international journal of experimental cellular physiology, biochemistry, and pharmacology. 2018;46(5):1951-70.
59. Li Y, Chen J, Chen XG, Wang L, Gautam SC, Xu YX, et al. Human marrow stromal cell therapy for stroke in rat: neurotrophins and functional recovery. Neurology. 2002;59(4):514-23.
60. Li Y, Chen J, Wang L, Lu M, Chopp M. Treatment of stroke in rat with intracarotid administration of marrow stromal cells. Neurology. 2001;56(12):1666-72.
61. Li Y, Chen J, Zhang CL, Wang L, Lu D, Katakowski M, et al. Gliosis and brain remodeling after treatment of stroke in rats with marrow stromal cells. Glia. 2005;49(3):407-17.
62. Li Y, Chopp M, Chen J, Wang L, Gautam SC, Xu YX, et al. Intrastriatal transplantation of bone marrow nonhematopoietic cells improves functional recovery after stroke in adult mice. J Cereb Blood Flow Metab. 2000;20(9):1311-9.
63. Li Y, McIntosh K, Chen J, Zhang C, Gao Q, Borneman J, et al. Allogeneic bone marrow stromal cells promote glial-axonal remodeling without immunologic sensitization after stroke in rats. Exp Neurol. 2006;198(2):313-25.
64. Lin X, Zhang Y, Liu W, Dong J, Lu J, Di Q, et al. Granulocyte-macrophage colony-stimulating factor-transfected bone marrow stromal cells for the treatment of ischemic stroke. Neural Regen Res. 2012;7(16):1220-7.

65. Liu H, Honmou O, Harada K, Nakamura K, Houkin K, Hamada H, et al. Neuroprotection by PlGF gene-modified human mesenchymal stem cells after cerebral ischaemia. Brain. 2006;129(Pt 10):2734-45.
66. Liu N, Chen R, Du H, Wang J, Zhang Y, Wen J. Expression of IL-10 and TNF-alpha in rats with cerebral infarction after transplantation with mesenchymal stem cells. Cell Mol Immunol. 2009;6(3):207-13.
67. Liu N, Zhang Y, Fan L, Yuan M, Du H, Cheng R, et al. Effects of transplantation with bone marrow-derived mesenchymal stem cells modified by Survivin on experimental stroke in rats. Journal of translational medicine. 2011;9:105.
68. Liu Z, Li Y, Qu R, Shen L, Gao Q, Zhang X, et al. Axonal sprouting into the denervated spinal cord and synaptic and postsynaptic protein expression in the spinal cord after transplantation of bone marrow stromal cell in stroke rats. Brain Res. 2007;1149:172-80.
69. Liu Z, Li Y, Zhang X, Savant-Bhonsale S, Chopp M. Contralesional axonal remodeling of the corticospinal system in adult rats after stroke and bone marrow stromal cell treatment. Stroke. 2008;39(9):2571-7.
70. Liu Z, Li Y, Zhang ZG, Cui X, Cui Y, Lu M, et al. Bone marrow stromal cells enhance inter- and intracortical axonal connections after ischemic stroke in adult rats. J Cereb Blood Flow Metab. 2010;30(7):1288-95.
71. Lu H, Liu X, Zhang N, Zhu X, Liang H, Sun L, et al. Neuroprotective Effects of Brain-Derived Neurotrophic Factor and Noggin-Modified Bone Mesenchymal Stem Cells in Focal Cerebral Ischemia in Rats. Journal of stroke and cerebrovascular diseases : the official journal of National Stroke Association. 2016;25(2):410-8.
72. Lu SS, Liu S, Zu QQ, Xu XQ, Yu J, Wang JW, et al. In vivo MR imaging of intraarterially delivered magnetically labeled mesenchymal stem cells in a canine stroke model. PloS one. 2013;8(2):e54963.
73. Ma S, Zhong D, Chen H, Zheng Y, Sun Y, Luo J, et al. The immunomodulatory effect of bone marrow stromal cells (BMSCs) on interleukin (IL)-23/IL-17-mediated ischemic stroke in mice. J Neuroimmunol. 2013;257(1-2):28-35.
74. Miki Y, Nonoguchi N, Ikeda N, Coffin RS, Kuroiwa T, Miyatake S. Vascular endothelial growth factor gene-transferred bone marrow stromal cells engineered with a herpes simplex virus type 1 vector can improve neurological deficits and reduce infarction volume in rat brain ischemia. Neurosurgery. 2007;61(3):586-94; discussion 94-5.
75. Mimura T, Dezawa M, Kanno H, Yamamoto I. Behavioral and histological evaluation of a focal cerebral infarction rat model transplanted with neurons induced from bone marrow stromal cells. J Neuropathol Exp Neurol. 2005;64(12):1108-17.
76. Mitkari B, Nitzsche F, Kerkela E, Kuptsova K, Huttunen J, Nystedt J, et al. Human bone marrow mesenchymal stem/stromal cells produce efficient localization in the brain and enhanced angiogenesis after intra-arterial delivery in rats with cerebral ischemia, but this is not translated to behavioral recovery. Behavioural brain research. 2014;259:50-9.
77. Miyamoto M, Kuroda S, Zhao S, Magota K, Shichinohe H, Houkin K, et al. Bone marrow stromal cell transplantation enhances recovery of local glucose metabolism after cerebral infarction in rats: a serial 18F-FDG PET study. Journal of nuclear medicine : official publication, Society of Nuclear Medicine. 2013;54(1):145-50.
78. Moisan A, Favre I, Rome C, De Fraipont F, Grillon E, Coquery N, et al. Intravenous Injection of Clinical Grade Human MSCs After Experimental Stroke: Functional Benefit and Microvascular Effect. Cell transplantation. 2016;25(12):2157-71.
79. Nam HS, Kwon I, Lee BH, Kim H, Kim J, An S, et al. Effects of Mesenchymal Stem Cell Treatment on the Expression of Matrix Metalloproteinases and Angiogenesis during Ischemic Stroke Recovery. PloS one. 2015;10(12):e0144218.

80. Nomura T, Honmou O, Harada K, Houkin K, Hamada H, Kocsis JD. I.V. infusion of brain-derived neurotrophic factor gene-modified human mesenchymal stem cells protects against injury in a cerebral ischemia model in adult rat. Neuroscience. 2005;136(1):161-9.
81. Okazaki T, Magaki T, Takeda M, Kajiwara Y, Hanaya R, Sugiyama K, et al. Intravenous administration of bone marrow stromal cells increases survivin and Bcl-2 protein expression and improves sensorimotor function following ischemia in rats. Neurosci Lett. 2008;430(2):109-14.
82. Omori Y, Honmou O, Harada K, Suzuki J, Houkin K, Kocsis JD. Optimization of a therapeutic protocol for intravenous injection of human mesenchymal stem cells after cerebral ischemia in adult rats. Brain Res. 2008;1236:30-8.
83. Onda T, Honmou O, Harada K, Houkin K, Hamada H, Kocsis JD. Therapeutic benefits by human mesenchymal stem cells (hMSCs) and Ang-1 gene-modified hMSCs after cerebral ischemia. J Cereb Blood Flow Metab. 2008;28(2):329-40.
84. Osanai T, Kuroda S, Yasuda H, Chiba Y, Maruichi K, Hokari M, et al. Noninvasive transplantation of bone marrow stromal cells for ischemic stroke: preliminary study with a thermoreversible gelation polymer hydrogel. Neurosurgery. 2010;66(6):1140-7; discussion 7.
85. Pavlichenko N, Sokolova I, Vijde S, Shvedova E, Alexandrov G, Krouglyakov P, et al. Mesenchymal stem cells transplantation could be beneficial for treatment of experimental ischemic stroke in rats. Brain Res. 2008;1233:203-13.
86. Pirzad Jahromi G, Seidi S, Sadr SS, Shabanzadeh AP, Keshavarz M, Kaka GR, et al. Therapeutic effects of a combinatorial treatment of simvastatin and bone marrow stromal cells on experimental embolic stroke. Basic Clin Pharmacol Toxicol. 2012;110(6):487-93.
87. Pirzad Jahromi G, Shabanzadeh Pirsaraei A, Sadr SS, Kaka G, Jafari M, Seidi S, et al. Multipotent bone marrow stromal cell therapy promotes endogenous cell proliferation following ischemic stroke. Clinical and experimental pharmacology & physiology. 2015;42(11):1158-67.
88. Pourheydar B, Soleimani Asl S, Azimzadeh M, Rezaei Moghadam A, Marzban A, Mehdizadeh M. Neuroprotective Effects of Bone Marrow Mesenchymal Stem Cells on Bilateral Common Carotid Arteries Occlusion Model of Cerebral Ischemia in Rat. Behavioural neurology. 2016;2016:2964712.
89. Ren W, Ma X, Liu X, Li Y, Jiang Z, Zhao Y, et al. Moderate hypothermia induces protein SUMOylation in bone marrow stromal cells and enhances their tolerance to hypoxia. Molecular medicine reports. 2017;16(5):7006-12.
90. Saito H, Magota K, Zhao S, Kubo N, Kuge Y, Shichinohe H, et al. 123I-iomazenil single photon emission computed tomography visualizes recovery of neuronal integrity by bone marrow stromal cell therapy in rat infarct brain. Stroke. 2013;44(10):2869-74.
91. Sammali E, Alia C, Vegliante G, Colombo V, Giordano N, Pischiutta F, et al. Intravenous infusion of human bone marrow mesenchymal stromal cells promotes functional recovery and neuroplasticity after ischemic stroke in mice. Scientific reports. 2017;7(1):6962.
92. Sasaki M, Honmou O, Radtke C, Kocsis JD. Development of a middle cerebral artery occlusion model in the nonhuman primate and a safety study of i.v. infusion of human mesenchymal stem cells. PloS one. 2011;6(10):e26577.
93. Sasaki Y, Sasaki M, Kataoka-Sasaki Y, Nakazaki M, Nagahama H, Suzuki J, et al. Synergic Effects of Rehabilitation and Intravenous Infusion of Mesenchymal Stem Cells After Stroke in Rats. Physical therapy. 2016;96(11):1791-8.
94. Shen LH, Chen J, Shen HC, Ye M, Liu XF, Ding WS, et al. Possible Mechanism of Therapeutic Effect of 3-Methyl-1-phenyl-2-pyrazolin-5-one and Bone Marrow Stromal Cells Combination Treatment in Rat Ischemic Stroke Model. Chinese medical journal. 2016;129(12):1471-6.
95. Shen LH, Li Y, Chen J, Cui Y, Zhang C, Kapke A, et al. One-year follow-up after bone marrow stromal cell treatment in middle-aged female rats with stroke. Stroke. 2007;38(7):2150-6.

96. Shen LH, Li Y, Chen J, Zacharek A, Gao Q, Kapke A, et al. Therapeutic benefit of bone marrow stromal cells administered 1 month after stroke. J Cereb Blood Flow Metab. 2007;27(1):6-13.
97. Shen LH, Li Y, Chen J, Zhang J, Vanguri P, Borneman J, et al. Intracarotid transplantation of bone marrow stromal cells increases axon-myelin remodeling after stroke. Neuroscience. 2006;137(2):393-9.
98. Shen LH, Xin H, Li Y, Zhang RL, Cui Y, Zhang L, et al. Endogenous tissue plasminogen activator mediates bone marrow stromal cell-induced neurite remodeling after stroke in mice. Stroke. 2011;42(2):459-64.
99. Shen LH, Ye M, Ding XS, Han Q, Zhang C, Liu XF, et al. Protective effects of MCI-186 on transplantation of bone marrow stromal cells in rat ischemic stroke model. Neuroscience. 2012;223:315-24.
100. Shichinohe H, Kuroda S, Sugiyama T, Ito M, Kawabori M. Bone marrow stromal cell transplantation attenuates cognitive dysfunction due to chronic cerebral ischemia in rats. Dement Geriatr Cogn Disord. 2010;30(4):293-301.
101. Shichinohe H, Kuroda S, Yano S, Hida K, Iwasaki Y. Role of SDF-1/CXCR4 system in survival and migration of bone marrow stromal cells after transplantation into mice cerebral infarct. Brain Res. 2007;1183:138-47.
102. Shichinohe H, Yamauchi T, Saito H, Houkin K, Kuroda S. Bone marrow stromal cell transplantation enhances recovery of motor function after lacunar stroke in rats. Acta Neurobiol Exp (Wars). 2013;73(3):354-63.
103. Song M, Lee JH, Bae J, Bu Y, Kim EC. Human Dental Pulp Stem Cells Are More Effective Than Human Bone Marrow-Derived Mesenchymal Stem Cells in Cerebral Ischemic Injury. Cell transplantation. 2017;26(6):1001-16.
104. Song M, Mohamad O, Gu X, Wei L, Yu SP. Restoration of intracortical and thalamocortical circuits after transplantation of bone marrow mesenchymal stem cells into the ischemic brain of mice. Cell transplantation. 2013;22(11):2001-15.
105. Steiner B, Roch M, Holtkamp N, Kurtz A. Systemically administered human bone marrow-derived mesenchymal stem home into peripheral organs but do not induce neuroprotective effects in the MCAo-mouse model for cerebral ischemia. Neurosci Lett. 2012;513(1):25-30.
106. Suda S, Shimazaki K, Ueda M, Inaba T, Kamiya N, Katsura K, et al. Combination therapy with bone marrow stromal cells and FK506 enhanced amelioration of ischemic brain damage in rats. Life Sci. 2011;89(1-2):50-6.
107. Sugiyama T, Kuroda S, Takeda Y, Nishio M, Ito M, Shichinohe H, et al. Therapeutic impact of human bone marrow stromal cells expanded by animal serum-free medium for cerebral infarct in rats. Neurosurgery. 2011;68(6):1733-42; discussion 42.
108. Suzuki J, Sasaki M, Harada K, Bando M, Kataoka Y, Onodera R, et al. Bilateral cortical hyperactivity detected by fMRI associates with improved motor function following intravenous infusion of mesenchymal stem cells in a rat stroke model. Brain Res. 2013;1497:15-22.
109. Tan C, Zhao S, Higashikawa K, Wang Z, Kawabori M, Abumiya T, et al. [(18)F]DPA-714 PET imaging shows immunomodulatory effect of intravenous administration of bone marrow stromal cells after transient focal ischemia. EJNMMI research. 2018;8(1):35.
110. Toyama K, Honmou O, Harada K, Suzuki J, Houkin K, Hamada H, et al. Therapeutic benefits of angiogenetic gene-modified human mesenchymal stem cells after cerebral ischemia. Exp Neurol. 2009;216(1):47-55.
111. Ukai R, Honmou O, Harada K, Houkin K, Hamada H, Kocsis JD. Mesenchymal stem cells derived from peripheral blood protects against ischemia. J Neurotrauma. 2007;24(3):508-20.
112. van Velthoven CT, Sheldon RA, Kavelaars A, Derugin N, Vexler ZS, Willemen HL, et al. Mesenchymal stem cell transplantation attenuates brain injury after neonatal stroke. Stroke. 2013;44(5):1426-32.

113. Wang C, Li F, Guan Y, Zhu L, Fei Y, Zhang J, et al. Bone marrow stromal cells combined with oxiracetam influences the expression of B-cell lymphoma 2 in rats with ischemic stroke. Journal of stroke and cerebrovascular diseases : the official journal of National Stroke Association. 2014;23(10):2591-7.
114. Wang LQ, Lin ZZ, Zhang HX, Shao B, Xiao L, Jiang HG, et al. Timing and dose regimens of marrow mesenchymal stem cell transplantation affect the outcomes and neuroinflammatory response after ischemic stroke. CNS Neurosci Ther. 2014;20(4):317-26.
115. Wang T, Tang W, Sun S, Xu T, Wang H, Guan J, et al. Intravenous infusion of bone marrow mesenchymal stem cells improves brain function after resuscitation from cardiac arrest. Crit Care Med. 2008;36(11 Suppl):S486-91.
116. Wang Y, Deng Y, Zhou GQ. SDF-1alpha/CXCR4-mediated migration of systemically transplanted bone marrow stromal cells towards ischemic brain lesion in a rat model. Brain Res. 2008;1195:104-12.
117. Wang Y, Geng T, Ni A, Yin H, Han B. Effects of transplanted GDNF gene modified marrow stromal cells on focal cerebral ischemia in rats. Front Integr Neurosci. 2011;5:89.
118. Wei L, Fraser JL, Lu ZY, Hu X, Yu SP. Transplantation of hypoxia preconditioned bone marrow mesenchymal stem cells enhances angiogenesis and neurogenesis after cerebral ischemia in rats. Neurobiol Dis. 2012;46(3):635-45.
119. Wei N, Yu SP, Gu X, Taylor TM, Song D, Liu XF, et al. Delayed intranasal delivery of hypoxic-preconditioned bone marrow mesenchymal stem cells enhanced cell homing and therapeutic benefits after ischemic stroke in mice. Cell transplantation. 2013;22(6):977-91.
120. Wu J, Sun Z, Sun HS, Wu J, Weisel RD, Keating A, et al. Intravenously administered bone marrow cells migrate to damaged brain tissue and improve neural function in ischemic rats. Cell transplantation. 2008;16(10):993-1005.
121. Xu Y, Du S, Yu X, Han X, Hou J, Guo H. Human bone marrow mesenchymal stem cell transplantation attenuates axonal injury in stroke rats. Neural Regen Res. 2014;9(23):2053-8.
122. Yamauchi T, Kuroda Y, Morita T, Shichinohe H, Houkin K, Dezawa M, et al. Therapeutic effects of human multilineage-differentiating stress enduring (MUSE) cell transplantation into infarct brain of mice. PloS one. 2015;10(3):e0116009.
123. Yan F, Yue W, Zhang YL, Mao GC, Gao K, Zuo ZX, et al. Chitosan-collagen porous scaffold and bone marrow mesenchymal stem cell transplantation for ischemic stroke. Neural Regen Res. 2015;10(9):1421-6.
124. Yan T, Venkat P, Chopp M, Zacharek A, Ning R, Roberts C, et al. Neurorestorative Responses to Delayed Human Mesenchymal Stromal Cells Treatment of Stroke in Type 2 Diabetic Rats. Stroke. 2016;47(11):2850-8.
125. Yan T, Ye X, Chopp M, Zacharek A, Ning R, Venkat P, et al. Niaspan attenuates the adverse effects of bone marrow stromal cell treatment of stroke in type one diabetic rats. PloS one. 2013;8(11):e81199.
126. Yang M, Wei X, Li J, Heine LA, Rosenwasser R, Iacovitti L. Changes in host blood factors and brain glia accompanying the functional recovery after systemic administration of bone marrow stem cells in ischemic stroke rats. Cell transplantation. 2010;19(9):1073-84.
127. Yang Z, Cai X, Xu A, Xu F, Liang Q. Bone marrow stromal cell transplantation through tail vein injection promotes angiogenesis and vascular endothelial growth factor expression in cerebral infarct area in rats. Cytotherapy. 2015;17(9):1200-12.
128. Ye Y, Peng YR, Hu SQ, Yan XL, Chen J, Xu T. In Vitro Differentiation of Bone Marrow Mesenchymal Stem Cells into Neuron-Like Cells by Cerebrospinal Fluid Improves Motor Function of Middle Cerebral Artery Occlusion Rats. Frontiers in neurology. 2016;7:183.
129. Yoo SW, Kim SS, Lee SY, Lee HS, Kim HS, Lee YD, et al. Mesenchymal stem cells promote proliferation of endogenous neural stem cells and survival of newborn cells in a rat stroke model. Exp Mol Med. 2008;40(4):387-97.

130. Zacharek A, Shehadah A, Chen J, Cui X, Roberts C, Lu M, et al. Comparison of bone marrow stromal cells derived from stroke and normal rats for stroke treatment. Stroke. 2010;41(3):524-30.
131. Zhang C, Li Y, Chen J, Gao Q, Zacharek A, Kapke A, et al. Bone marrow stromal cells upregulate expression of bone morphogenetic proteins 2 and 4, gap junction protein connexin-43 and synaptophysin after stroke in rats. Neuroscience. 2006;141(2):687-95.
132. Zhang H, Sun F, Wang J, Xie L, Yang C, Pan M, et al. Combining Injectable Plasma Scaffold with Mesenchymal Stem/Stromal Cells for Repairing Infarct Cavity after Ischemic Stroke. Aging and disease. 2017;8(2):203-14.
133. Zhang HL, Xie XF, Xiong YQ, Liu SM, Hu GZ, Cao WF, et al. Comparisons of the therapeutic effects of three different routes of bone marrow mesenchymal stem cell transplantation in cerebral ischemic rats. Brain Res. 2018;1680:143-54.
134. Zhang J, Li Y, Chen J, Yang M, Katakowski M, Lu M, et al. Expression of insulin-like growth factor 1 and receptor in ischemic rats treated with human marrow stromal cells. Brain Res. 2004;1030(1):19-27.
135. Zhang Q, Chen ZW, Zhao YH, Liu BW, Liu NW, Ke CC, et al. Bone Marrow Stromal Cells Combined With Sodium Ferulate and n-Butylidenephthalide Promote the Effect of Therapeutic Angiogenesis via Advancing Astrocyte-Derived Trophic Factors After Ischemic Stroke. Cell transplantation. 2017;26(2):229-42.
136. Zhang Q, Liu S, Li T, Yuan L, Liu H, Wang X, et al. Preconditioning of bone marrow mesenchymal stem cells with hydrogen sulfide improves their therapeutic potential. Oncotarget. 2016;7(36):58089-104.
137. Zhang Q, Zhao Y, Xu Y, Chen Z, Liu N, Ke C, et al. Sodium ferulate and n-butylidenephthalate combined with bone marrow stromal cells (BMSCs) improve the therapeutic effects of angiogenesis and neurogenesis after rat focal cerebral ischemia. Journal of translational medicine. 2016;14(1):223.
138. Zhang YX, Yuan MZ, Cheng L, Lin LZ, Du HW, Chen RH, et al. Treadmill exercise enhances therapeutic potency of transplanted bone mesenchymal stem cells in cerebral ischemic rats via anti-apoptotic effects. BMC neuroscience. 2015;16:56.
139. Zhao MZ, Nonoguchi N, Ikeda N, Watanabe T, Furutama D, Miyazawa D, et al. Novel therapeutic strategy for stroke in rats by bone marrow stromal cells and ex vivo HGF gene transfer with HSV-1 vector. J Cereb Blood Flow Metab. 2006;26(9):1176-88.
140. Zhao Y, Guan Y, Xu Y, Li Y, Wu W. Sodium Ferulate combined with bone marrow stromal cell treatment ameliorating rat brain ischemic injury after stroke. Brain Res. 2012;1450:157-65.
141. Zong X, Wu S, Li F, Lv L, Han D, Zhao N, et al. Transplantation of VEGF-mediated bone marrow mesenchymal stem cells promotes functional improvement in a rat acute cerebral infarction model. Brain Res. 2017;1676:9-18.
